# New insights into coordinated regulation of AHR promoter transcription; molecular mechanisms and therapeutic targets

**DOI:** 10.7150/ijbs.112869

**Published:** 2025-07-11

**Authors:** Kenly Wuputra, Chia-Che Ku, Wen-Hung Hsu, Tusty-Jiuan Hsieh, Yi-Chun Tsai, Chih-Yen Chen, Yoshiharu Tanaka, Ying-Chu Lin, Chao-Hung Kuo, Deng-Chyang Wu, Kazunari K. Yokoyama

**Affiliations:** 1 Cell Therapy Research Center, Department of Medicine, Kaohsiung Medical University Hospital, Kaohsiung 80756, Taiwan.; 2 Graduate Institute of Medicine, Kaohsiung Medical University, Kaohsiung 80708, Taiwan.; 3 Regenerative Medicine and Cell Research Center, Kaohsiung Medical University, Kaohsiung 80708, Taiwan.; 4 Division of Gastroenterology, Department of Internal Medicine, Kaohsiung Medical University Hospital, Kaohsiung 80756, Taiwan.; 5 Division of Gastroenterology, Department of Internal Medicine, Kaohsiung Medical University GangShan Hospital, Kaohsiung 820004, Taiwan.; 6 Division of Nephrology, Department of Internal Medicine, Kaohsiung Medical University Hospital, Kaohsiung 80756, Taiwan.; 7 Division of Nephrology, Department of Internal Medicine, Kaohsiung Medical University CiJin Hospital, Kaohsiung 805, Taiwan.; 8 Institute of Emergency and Critical Medicine, and School of Medicine, National Yang Ming Chiao Tung University, Taipei 112304, Taiwan.; 9 Division of Gastroenterology and Hepatology, Department of Medicine, Taipei Veterans General Hospital, Taipei 112201, Taiwan.; 10 Radiation Biology and Molecular Genetics, Division of Quantum Radiation, Faculty of Technology, Osaka Metropolitan University, Osaka 599-8531, Japan.; 11 School of Dentistry, Kaohsiung Medical University, Kaohsiung 80708, Taiwan.

**Keywords:** aryl hydrocarbon receptor, chromatin control, Jun dimerization protein, nuclear factor erythroid 2-related factor 2, reactive oxygen species, transcriptional regulation

## Abstract

The aryl hydrocarbon receptor (AHR) plays crucial roles in the control of stress, xenobiotic metabolism, inflammation, and cancer. However, information on the chromatin regulation of ligand-dependent *AHR* promoter activation is limited. AHR and nuclear factor erythroid 2-related factor 2 (NRF2) signaling are coordinated to maintain the balance of reactive oxygen species (ROS), which is termed the AHR-NRF2 gene battery. Recently, promoter activation of *AHR* to phase I ligands was reported to be regulated by AHR-NRF2-Jun dimerization protein 2 (JDP2) in a spatiotemporal manner. Tight coupling between phase I and II nuclear transcriptional factor complexes through histone chaperone JDP2 in a time- and space-dependent manner may occur in the chromatin to regulate phase I gene expression. This new mechanism, termed AHR-NRF2-JDP2 gene battery, may facilitate the identification of therapeutics at the reduction of reactive toxic intermediates at the nucleosome level. Identifying the AHR-NRF2-JDP2 gene battery mechanisms will enable the development of novel therapeutics for the risk assessment of oxidative stress/antioxidation, detoxification, ROS, cell death, inflammation, allergies, and cancer.

## 1. Introduction

The aryl hydrocarbon receptor (AHR) was identified as a possible receptor for the anthropogenic compound 2,7,8-tetrachlorodibenzo-p-dioxin (TCDD) in 1976 1. TCDD-bound AHR stimulates the expression of cytochrome P450 family 1 subfamily A member 1 (CYP1A1). Thus, the AHR has been identified as a ligand-activated transcription factor with physiological roles in health and disease 2-4. Studies in recombinant mice have indicated that AHR plays an important role in organ development as well as reproductive, hematopoietic, and immune response regulation 5,6. *AHR* consists of 11 exons and is located on chromosome 7p21 in humans and chromosome 12A3 in mice 7,8. The *AHR* promoter consists of a GC-rich sequence located near the transcription start site (TSS), which is bound by zinc finger transcription factors, such as SP1 and SP3, and lacks the TATA and CCAAT boxes 9-13.

Chromatin immunoprecipitation (ChIP) sequencing analysis of the AHR-aryl hydrocarbon receptor nuclear translocator (ARNT) complex has been used to identify the genes activated in response to TCDD. This complex was found to preferentially bind proximal promoter regions within 1 kb from the +1 TSS 14, indicating that the AHR target genes are mainly located within the proximal promoter. By contrast, the AHR/ARNT bound locus was also positioned distally (approximately 100 kb) from the annotated +1 site 15. This finding indicates that AHR regulates the proximal and distal promoters of the target genes. Notably, gene regulation occurs by remote *cis*-acting regions through chromatin remodeling, DNA looping, or even intra- and inter-chromosomal interactions 16. In addition, TCDD induced c-Jun and Jun D expression by the activation of AHR-ARNT through dioxin-responsive elements (DREs) or xenobiotic response elements (XREs) (core sequence: 5′-TA/TGCGTG-3′) in an AHR-dependent manner 17. These factors regulated AHR transcription in a cell- or cancer-type-specific manner 18. AHR expression was also regulated by the levels of epigenetic markers. Inhibitors of histone deacetylases (HDACs) increased, whereas histone acetyltransferase (HAT) inhibitors decreased AHR promoter activity. These findings indicate that histone acetylation changes in the epigenetic landscape are a critical regulator of AHR expression 19. Likewise, DNA hypermethylation induced by 5-aza-2′-deoxycytidine downregulated AHR expression in acute lymphoblastic leukemia (ALL) cell lines 20. Thus, epigenetic regulation, such as acetylation and methylation, is critical for AHR activation and its response to phase I ligands.

AHR transcription is initiated by the complex of phase I ligand-bound AHR with ARNT, which binds the XRE/DRE motif in the *AHR* promoter region as the canonical pathway of AHR activation 21-23. The phase I ligand binds to the AHR via the Per-Arnt-Sim (PAS) B domain and enables its translocation into the nucleus to induce AHR transcription via RNA polymerase II (Pol II). However, XRE/DRE elements, to which the AHR-ARNT complex binds, are also present in the promoter of the phase II enzyme transcription factor nuclear factor erythroid 2-related factor 2 (NRF2) 24.

Conversely, NRF2 along with musculoaponeurotic fibrosarcoma (sMAF) proteins directly activate the *AHR* promoter and AHR target genes, such as *CYP1A1* and *CYP1B1*, by recruiting the NRF2-sMAF complex to antioxidant response elements (AREs) in the gene promoters of *AHR*, *CYP1A1*, or *CYP1B1* detoxification phase I enzymes 25. The interconversion of phase I ligands, such as AHR ligands, activated phase I (AHR target gene promoters) or II enzyme gene promoters (NRF2 target gene promoters), such as NAD(P)H:quinone oxidoreductase 1 (*NQO-1*) and glutathione S-transferase alpha 1 gene promoters, which constituted the AHR-NRF2 gene battery 26-32.

The “gene battery” model was presented by Britten and Davidson in 1969 to elucidate the theoretical gene control for the regulated gene expression in eukaryotes 33. A gene battery is characterized as a group of nonlinked genes that exhibit cross talk, having an interrelationship regarding up- and downregulation, in response to some signal. The battery's response is mediated by certain regulatory proteins whose effect may be combinatorial in nature. For example, the mouse aromatic hydrocarbon-responsive gene battery was among the best-characterized examples of gene batteries in eukaryotes 34,35. Furthermore, because AHR agonists, such as TCDD, can stimulate the cross talk between the AHR/ARNT and NRF2/sMAF transcription factor complexes 25-32, the AHR-NRF2 gene battery was defined.

However, both AHR-ARNT and NRF2-sMAF complexes were found to bind different cis-elements in the *AHR* promoter to trigger the AHR transcription in a spatiotemporal manner in mouse embryonic fibroblasts (MEFs) in response to TCDD or dimethyl sulfoxide as the ligands 21,23. These phase I and II nuclear transcriptional factor complexes were associated with Jun dimerization protein 2 (JDP2), which was a chromatin modifier and histone chaperone 36,37. The coordinated activation of the *AHR* promoter and allele by the AHR-NRF2 axis, facilitated by the chromatin modifier JDP2 to open chromatin for activating RNA transcription and then close chromatin for terminating the RNA transcription of the *AHR* locus in a time- and space-dependent manner, was termed the AHR-NRF2-JDP2 gene battery. The chromatin regulation of the AHR-NRF2-JDP2 axis is summarized in **Table 1**. This new dogma indicates novel therapeutic targets for regulating oxidative stress-induced cell death, cell spreading, cellular metastasis, and inflammatory regulation against endogenous and exogenous stressors.

## 2. Genomic canonical pathway of AHR transcription

AHR is expressed in all tissues in humans and mice, with particularly high levels in the placenta, lung, kidney, liver, and thymus 38,39. The ligand- and nonligand-dependent pathways of AHR activation are independent. Nonligand-bound AHR was found to be present in the cytoplasm and formed an integrated complex with the heat shock protein 90 dimer, AHR-interacting protein (also named hepatitis B virus X-associated protein 2), cochaperone prostaglandin E synthase 3 (also known as p23), and the nonreceptor protein tyrosine kinase c-SRC (SRC) 40. On exposure to ligands, phase I ligand-induced AHR activation triggered the conformational change of the AHR complex in the cytoplasm and the release of AHR-interacting protein/hepatitis B virus X-associated protein 2 that exposed the nuclear localization signal, resulting in the translocation of this complex into the nucleus 41-43. In the nucleus, the heat shock protein 90 dimer, AHR-interacting protein, p23, and SRC dissociated from the AHR complex and the PAS domain of the AHR molecule and subsequently formed a transcription-competent complex with ARNT 44. The AHR-ARNT heterodimer initiated the expression of genes involved in xenobiotic metabolism, including phase I and II genes by recruiting RNA Pol II complexes to the DRE/XRE motifs in the promoters of these target genes 14-16,22. The phase I ligand bound to AHR via the PAS-B domain, and the ligand-AHR complex translocated into the nucleus to generate a transcription-competent complex with ARNT. This AHR/ARNT axis affects several biological processes, including inflammation, allergic responses, metabolism, genetic expression, infectious disease responses, neuronal diseases, cancer, and aging.

## 3. DNA methylation of the AHR locus

Methylation of the 5′-cytosine residues in CpG islands results in transcriptional repression 45. Methylation of the CpG islands (-33 to +174) of the *AHR* promoter in human ALL is responsible for *AHR* expression in a cell type-specific manner 20. The *AHR* promoter is hypermethylated and inactivated in ALL compared with normal cells 46. Demethylation and activation of the *AHR* promoter contribute to restoring the normal phenotype and blocking ALL induction. AHR expression is coordinated with the epigenetic regulation of DNA methylation enzymes, such as DNA methyltransferase 1 (DNMT1), DNMT3A, DNMT3B, and methyl binding protein 2. These molecules altered the histone methylation status of trimethylation of lysine 9 on the histone H3 protein (H3K9me3) in the breast cancer gene 1 (*BRCA1*) promoter, whereas the AHR inhibitor blocked the cross talk of AHR with methylation-associated signaling to activate BRCA1 expression 47-49. The cell type conversion is also dependent on ligand specificity and the expression of forkhead box P3 (FOXP3) on methylated CpG islands. Inhibition of AHR resulted in higher expression of FOXP3 and decreased methylation of CpG islands in the FOXP3 locus, where the binding of both DNMT1 and DNMT3B was reduced 50,51.

Thus, AHR activation is suggested to decrease the level of DNMT expression, indicating that AHR expression is correlated with the demethylation mediated by DNMTs.

The ten-eleven translocation 2 (TET2) promoter region contains cis-elements that can bind AHR complexed with ligands such as L-kynurenine (Kyn) 52. The AHR ligand promoted TET activation by inducing the promoter demethylation of ecto-5′-nucleotidase gene (also known as CD73), which converted adenosine monophosphate to adenosine. The repression of AHR was due to DNA methylation of the ecto-5′-nucleotidase gene promoter. This finding indicated that AHR contributed to the reduction of adenosine production in regulatory T cells or the B cells of systemic lupus erythematosus patients 53. AHR affected the histone modifications mediated by HDACs and promoted DNA demethylation through TET2 activation. Further studies are needed to investigate how AHR directly interacts with and alters epigenetic modifications and how these changes affect AHR and its target genes.

In liver cancer cells, AHR was found to be critical in base excision repair where methylated cytosine was replaced by nonmethylated cytosine in the *CYP1A1* promoter, leading to increased *CYP1A1* RNA expression 54. Moreover, activation-induced cytosine deaminase (AICDA=AID) was involved in the mRNA editing required for switching of the immunoglobulin isotype and somatic hypermutation in B cells. Deficiency in the *AICDA* gene led to a pure B-cell defect characterized by the absence of high-affinity antibodies and a significantly increased risk of infections 55. The targeting of DNMTs and CpG islands in the *AHR* promoter might aid the development of potential therapies for autoimmune arthritis.

## 4. Histone modifications of the *AHR* locus

Histone modifications, including acetylation, methylation, phosphorylation, ubiquitination, adenosine diphosphate ribosylation, and sumoylation, are regulators of gene expression 56. In addition, histone variants contribute to chromatin alterations 57 and epigenetic changes 58,59. Histone methyltransferases (HMTs) and protein arginine N-methyltransferases catalyze histone methylation, whereas histone demethylases mediate demethylation 60. HAT catalyzes the attachment of acetyl molecules to lysine residues on histones, whereas HDAC removes the acetyl groups on histones. The histone modification process is dynamic, and thus epigenetic transcription is regulated 56.

### 4.1. Acetylation and deacetylation of AHR/ARNT and NRF2

Regarding the acetylation of the AHR/ARNT complex, cyclic adenosine monophosphate response element-binding protein (CREB)-binding protein (CBP)/p300 interacted with ARNT or ARNT2 but not with AHR 61. Lysine acetyltransferases, including nuclear receptor coactivator 1 (NCOA1), NCOA3, and CBP/p300, were necessary for AHR-induced transcription of the *CYP1A1* gene 62. Weinert et al. demonstrated that AHR expression was repressed at both the transcript and protein levels in CBP/p300 knockout (KO) and acetyltransferase or bromodomain inhibitor-treated cells 63. Under normal conditions, CYP1A1 gene repression in MEFs was mediated by Aryl hydrocarbon receptor repressor (AHRR)/ARNT heterodimers, not by AHR/ARNT, and involved ankyrin repeat family A member 2, HDAC4, and HDAC5 as corepressors 64.

Epigenetic regulation of *AHR* transcriptional activation has been reported elsewhere 62,65. Tumor suppressor gene products can suppress *AHR* promoter activity. TCDD exposure induced the methylation of the promoter of the tumor suppressor genes p16^INK4a^ and p53 and subsequently repressed their transcription 66, indicating that the consensus sequences of DRE were important for ligand-bound AHR/ARNT complex. Moreover, other coactivators, such as CBP/p300 and TIP60, might play crucial roles in AHR/ARNT target gene expression via ARNT activity 62,65. Thus, the requirement for CBP/p300-catalyzed acetylation in the AHR-dependent pathway is still unclear. Further studies are required to address this issue.

Regarding epigenetic modulation by NRF2, CBP/p300 directly acetylated NRF2 in response to arsenic exposure, and several acetylated lysine residues within the Neh1 domain (DNA-binding domain) of NRF2 interacted with CBP/p300 67. Thus, the acetylation of NRF2 by CBP increased the promoter-specific DNA-binding activity of NRF2 and enhanced NRF2-mediated antioxidant responses 68. Both HATs and HDACs regulated the acetylation levels of NRF2. Acetylation was found in multiple functional domains of NRF2, particularly within the transactivation domain and other critical structural domains 69. The overexpression of N-α-acetyltransferase 10 in colorectal cancer and the histone acetyltransferase (males absent on the first; MOF =KAT8) in non-small cell lung cancer enhanced NRF2 acetylation and nuclear localization to induce the respective NRF2 target genes for cancer progression 70,71. By contrast, HDAC3 was involved in NRF2-mediated pulmonary fibrosis 72. HDAC5 inhibited NRF2-dependent antioxidant genes in cardiomyocytes 73. Inhibition of HDAC6 protected mice from experimental stroke-induced brain injury 74. Moreover, the epigenetic modification of NRF2 was summarized recently in separate reviews 75,76.

### 4.2. Acetylation and deacetylation of AHR target genes

Inhibitors of HATs and HDACs can block specific histone codes for transcription, including that of *AHR*. For example, butyrate as an HDAC inhibitor increased AHR recruitment to the target gene promoter in response to a tryptophan-derived AHR agonist 77.

#### 4.2.1. HDAC1/RHO-A/HIF/pRB2/p53

The effects of the inhibitor of 3-hydroxy-3-methylglutaryl coenzyme A reductase, simvastatin, on tumor induction mediated by 3-methylcholanthrene (3MC) were examined in human renal epithelial cells. The increased expression of HDAC1 and decreased expression of RAS homolog family member A (RHO-A) were found through hypoxia-inducible factor- and AHR-dependent pathway 78. 3MC reduced the cell growth by the epigenetic modification of histones through an AhR/RhoA-dependent mechanism that could be reversed using statins (or HMG-CoA reductase inhibitors), which can inhibit Rho. Thus, Statins reversed the effect of 3MC to inhibit DNA synthesis by decreasing the nuclear translocation of the pRb2/HDAC1 complex, leading to a recovery of the levels of cell-cycle regulatory proteins 79,80. AHR upregulated Rb2 and HDAC1, which inhibited the growth of 3MC-treated vascular endothelial cells 79. Overexpression of HDAC1 led to poor survival in tumor cells 81, whereas HDAC1 knockdown inhibited progression through the G2/M checkpoint of the cell cycle and suppressed the proliferation of cancer cells, resulting in p53 deacetylation, which inhibited p53-mediated cell death 82.

#### 4.2.2 HDAC8/pRB1

Deletion of HDAC8 has been shown to increase Structural maintenance of chromosomes 3 (SMC3) acetylation and the inefficient dissolution of cohesin complexes 83. In addition, the mechanism linking AHR and hepatocellular carcinomas via HDAC8, which promoted tumor cell growth and may restrain the expression of retinoblastoma 1 (RB1) tumor suppressor 84.

#### 4.2.3. HDAC2/LTBP-1

Moreover, gut-microbiota infection and HDAC inhibition by butyrate or valproic acid both regulated AHR expression during immune surveillance and inflammation reactions 77,85-87. Furthermore, HDAC2 bound to the latent transforming growth factor-β-binding protein 1 (LTBP-1) promoter, leading to the inhibition of its expression in wild-type MEFs, whereas the HDAC2 deficiency and the binding of phosphorylated CREB (Ser133) enabled the activation of LTBP-1 transcription in AHR^-/-^ MEFs. Thus, epigenetic regulation can contribute to inhibiting constitutive LTBP-1 expression mediated by AHR 88.

#### 4.2.4. HDAC1/PI3K/AKT/HIF1α

HDAC inhibitors, including sodium butyrate and curcumin, reduced oxidative stress production and airway inflammation in asthmatic mice by inhibiting HDAC1 through phosphoinositide 3-kinase (PI3K)/AKT/hypoxia-inducible factor-1α/vascular endothelial growth factor signaling 89.

#### 4.2.5. AHRR/CYP1A2/SIRT3/SIRT7

Moreover, indoxyl sulfate, which was an AHR agonist/L-tryptophan metabolite, regulated the expression of AHRR, CYP1A2, sirtuin-3 (SIRT3), and SIRT7 to induce DNA damage and affect bone mineral status 90. In addition, butyrate acted as an HDAC inhibitor leading to increased AHR recruitment to the target gene promoters in response to tryptophan-derived AHR agonists. These findings suggested a novel understanding of AHR regulation mediated by an interaction between the gut and microbiota-derived metabolites 77.

### 4.3. Histone modifications of AHR target epigenetic landmarks

H3K4me1 is a hallmark of transcriptional enhancers 91, whereas H3K4me3 is highly enriched at TSSs 78. In addition, the modification H3K36me3 mediated by the Histone methyltransferases (HMT) Su(var)3-9, Enhancer-of-zeste and Trithorax (SET) domain containing two proteins suppressed cryptic transcription, regulated splicing reactions, and served as a binding site for transcriptional elongation factors 92. H3K79me2 positively correlated with the genetic program of male germ cells throughout spermatogenesis. The HMT Disruptor of telomeric silencing 1-like (DOT1L), which generates the H3K79me2 modification, predominantly mediated gene repression rather than activation 93. H3K79me is associated with active chromatin and transcriptional regulation, whereas H3K9me2 and H3K27me3 are typically found in closed, silenced chromatin regions 94. By contrast, H3K9ac and H3K27ac are often associated with enhancers and promoters of active genes 95. Both H3K14ac and H4K16ac promote chromatin opening, which facilitates the recruitment of transcriptional machinery to DNA 96. Phosphorylation of H3S10, H3S28, and H2AT120 is involved in regulating chromatin status during mitosis 97. Moreover, the phosphorylation of H2AXS139 (γ-H2AX) acts as a signal for the recruitment of DNA repair proteins 98.

The histone modification and acetylation modes of each histone of the AHR-NRF2-JDP2 complex have not been reported in detail, except the finding that JDP2 as a histone chaperone interacted with all histone species and inhibited p300-mediated histone acetylation at H4K8ac and H4K16ac, but not at H4K5ac and H4K12ac 36. Thus, further studies are required to define the interaction of histones with this complex. One key question is how these histone modifications specifically relate to AHR expression and function. Therefore, we describe below the series of histone changes in the context of specific ligands in AHR regulation. AHR affects local histone acetylation/methylation by interacting with coactivators or displacing HDAC complexes or corepressors 99.

#### 4.3.1. H3KK9ac, H3K14ac, H3K27ac, H3K4me1/2/3, H3K9me1/2/3, and H3K27me, HDAC2, HDAC4

Environmental toxicants have been reported to induce neurological anomalies and cancers through histone modifications, because investigating the underlying key physiological and pathological pathways is important regarding human health 100. Most prominent toxicants, such as bisphenol A, heavy metals, pesticides, and phthalates, are responsible for neurological impairments caused by epigenetic modifications via the alteration of histone-modifying enzymes, such as HATs, HDACs, and HMTs. These enzymes mediated chromatin remodeling; HATs and HMTs attenuated the expression of certain histone modifications, including H3K9ac, H3K14ac, H3K27ac, H3K4me1/2/3, H3K9me1/2/3, and H3K27me, whereas the amplification of HDAC2 and HDAC4 collectively altered the gene expression of certain proteins that regulated vital molecular pathways, including AHR.

#### 4.3.2. H3K9me2 and H3K9me3

Exposure to arsenic and benzo[a]pyrene (BaP) synergistically induced cellular transformation and tumorigenesis to promote lung tumorigenesis 88. The histone-lysine N-methyltransferase SUV39H1 trimethylated lysine 9 of histone H3 (H3K9me3). H3K9me2 levels were regulated by SUV39H1 and enriched in the promoter of the suppressor of cytokine signaling 3 gene in cells with arsenic and BaP co-exposure compared with those in cells with BaP exposure alone.

#### 4.3.3. H3K4me2

Depletion of an orphan nuclear receptor NR2E3 promoted the recruitment of lysine-specific histone demethylase-1, which decreased H3K4me2 levels and subsequently decreased *AHR* transcription 89. Ahr and H3K4me2 levels were reduced significantly in the livers of Nr2e3^rd7/rd7^ mice with a loss of NR2E3. Treatment with lysine-specific histone demethylase-1 inhibitors led to an increase in AhR and H3K4me2 levels in Rd7 mice. In addition, the AhR-depleted mice showed an increased frequency of diethylnitrosamine-induced liver tumors.

#### 4.3.4. MALT1/EZH2/H3K27me3

TCDD exposure induced a long noncoding RNA, metastasis associated in lung adenocarcinoma transcript-1 (MALAT1) in AsPC-1 and PANC-1 cancer cells 90. AhR transcriptionally upregulated MALAT1, which concomitantly increased the level of EZH2 to increase the levels of H3K27me3. TCDD exposure resulted in a significant increase in MALAT1, EZH2, and H3K27me3 levels but exposure to AhR antagonists exhibited the reversed functions of MALAT1, EZH2, and H3K27me3 in AhR-overexpressing pancreatic cancer cells.

#### 4.3.5. H4K5ac, H4K8ac, H4K12ac, H4K16ac, MAT2

The tryptophan metabolite cinnabarinic acid (CA) was an endogenous activator of AhR that failed to induce hepatic Cyp1a1 but upregulated a novel AhR target gene, a peptide hormone called stanniocalcin 2 (Stc2) in the liver 91. CA-dependent AhR-XRE-mediated Stc2 upregulation was responsible for cytoprotection against endoplasmic reticulum/oxidative stress-induced apoptosis. This AHR activation was mediated by CA but not by TCDD. In this selective response mechanism, the complex between AHR/ARNT and metastasis tumor-associated protein 2 (MTA2) was a component of the nucleosome remodeling and deacetylase (NURD) complex. MTA2 recruitment was required for the acetylation of H4K5, H4K8, H4K12, and H4K16. This finding is interesting because MTA2 is a chromatin-modifying protein and a component of the NURD complex. Thus, MTA2 may regulate both the repression and activation of gene expression 92.

#### 4.3.6. H3K4me4 and H4K20me3

Dioxin induced AHR-dependent DNA demethylation of the *CYP1A1* promoter in the mouse liver, which led to an increase in H3K4me3 levels and a significant decrease in H4K20me3 levels 54.

#### 4.3.7. H4K4ac, H3K9ac, and H3K9me

Resveratrol mediated the reverse epigenetic changes associated with AHR activation and its binding to the *BRCA1* promoter in breast cancer cells 48,49,101. The activation and recruitment of AHR to the *BRCA1* promoter hampered 17β-estradiol-induced activation of *BRCA1* transcription. These inhibitory effects were accompanied by a reduction in estrogen receptor alpha occupancy and histone H4K4Ac and H3K9Ac levels. Conversely, TCDD increased the association of H3K9me, DNMT1, and methyl-CpG binding domain protein 2 with the *BRCA1* promoter and promoted the accumulation of DNA strand breaks. The AHR-dependent repression of BRCA1 expression was reversed by the silencing of *AHR* and *DNMT1* by small interfering RNAs or pretreatment with resveratrol, which inhibited the DNA double-strand breaks induced by TCDD.

#### 4.3.8. H3K14ac, H4K16ac, H3K4me3, and H3S10p

CYP1A1 activation by AHR/ARNT was concerned with specific chromatin marks, including H3K14ac, H4K16ac, H3K4me3, and phosphorylation of H3S10. The complex of HDAC1 and DNMT1 was formed on the *CYP1A1* promoter of uninduced cells. However, HDAC1 inhibition alone was not sufficient to induce CYP1A1 expression, although it enabled the hyperacetylation of H3K14 and H4K16 to levels similar to those found in BaP-treated cells. These findings indicated that HDAC1 inhibition was necessary but insufficient for *CYP1A1* induction 94.

#### 4.3.9. H1K34hcit

TCDD-activated AHR dimerized with KLF6 and carbamoyl phosphate synthetase 1 and bound to the non-consensus XRE. The recruitment of carbamoyl phosphate synthetase 1 resulted in the localized synthesis of carbamoyl phosphate and histone H1 homo-citrullination (H1K34hcit) in an enzyme-independent manner. H1K34hcit represents a hitherto unknown epigenetic mark implicated in enhanced gene expression of the peptidyl arginine deiminase 2 gene, which itself is a chromatin-modifying protein 102.

## 5. Nongenomic pathways of AHR transcription

The nongenomic pathways of AHR transcription have been summarized previously 103-105. AHR can interact with signaling pathways involving epidermal growth factor receptor kinase, focal adhesion kinase, mitogen-activated protein kinase (RAS/RAF/MEK1/2/ERK1/2 and PI3K/AKT/mTOR pathways), protein kinase C, signal transducer and activator of transcription, SRC, and NF-κB.

## 5. AHR-NRF2 gene battery

### 5.1. Mechanism of TCDD-induced AHR promoter activation

**Figure 1:**
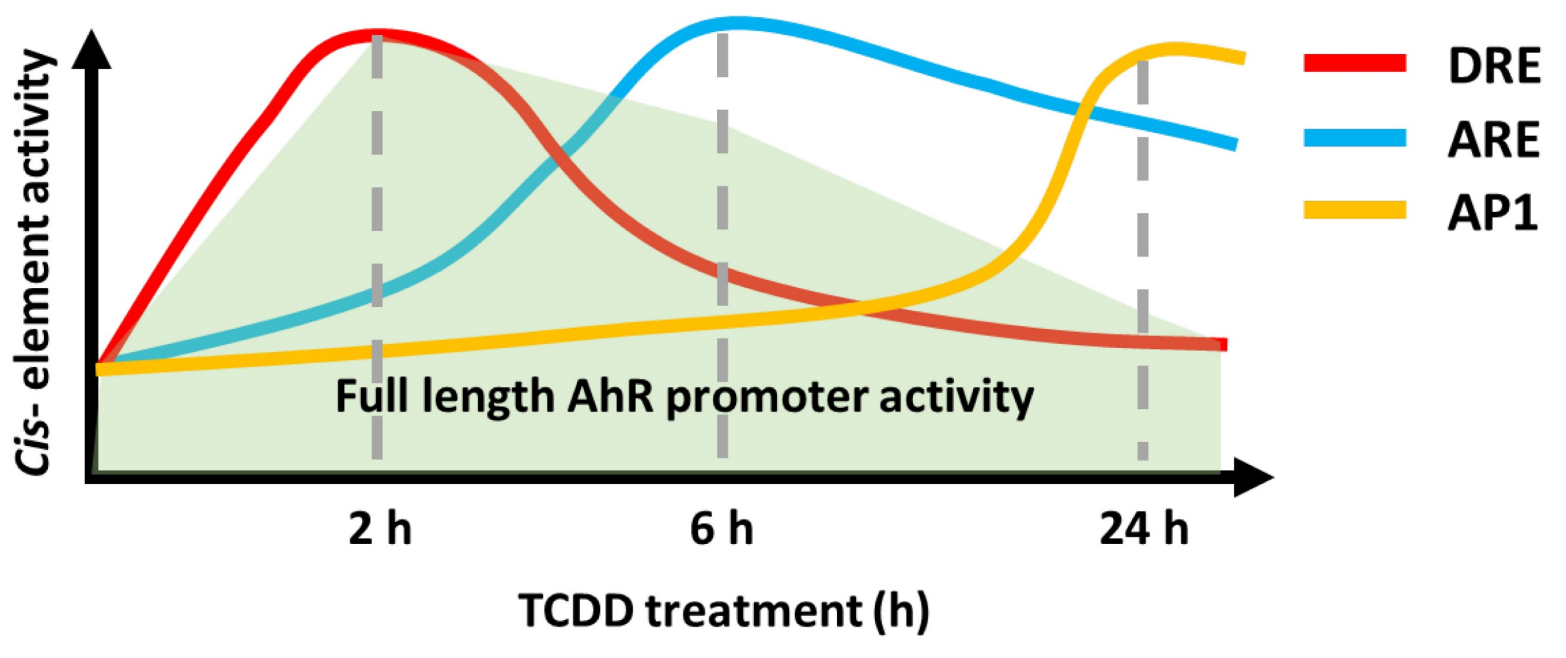
** Time course of promoter activity of DRE-, ARE-, and AP-1 luciferase in wild-type MEFs in response to TCDD.** Wild-type MEFs were incubated with 10 nM TCDD, a phase I enzyme ligand, and the luciferase activity was measured at each time point using DRE-luciferase (red line), ARE-luciferase (blue line), AP-1 luciferase (brown line), and AHR-luciferase (light green) as described elsewhere 21. The schematic model represents the time course of each *cis*-element mutated luciferase as described elsewhere 21.

Induction of the AHR-JDP2-NRF2 axis by TCDD is a time-ordered process, with the following three key stages: DRE response, ARE response, and AP-1 response. This time-dependent regulation of the AHR-NRF2-JDP2 complex occurs by exposure to phase I enzyme ligands, such as TCDD, 6-formylindolo[3,2-*b*] carbazole, BaP, and Kyn. By contrast, this ligand-specific promoter activity was repressed in *Jdp2^-^*^/^*^-^* MEFs 21. Thus, these regulatory mechanisms appeared to be dependent on the phase I ligands. In addition, the time course of TCDD exposure in MEFs containing DRE-, ARE-, and AHR-luciferase constructs as well as each cis-element mutant DRE2/3, ARE1 and AP-1 of AHR-luciferase confirmed that the regulation of TCDD-induced *AHR* promoter activation was time- and space-dependent (**Fig. 1**).

In wild-type MEFs treated with TCDD, the response of *AHR* promoter activation was typically initiated at 2-6 h after TCDD stimulation. Furthermore, TCDD-bound AHR can associate with JDP2-associated chromatin modulators, such as the cohesion complex and switch/sucrose nonfermentable (SWI/SNF2) complex including brahma-related gene 1 (BRG1) through mediators (MEDs) to open the closed chromatin and direct the Pol II transcription initiation complex to the DREs (unpublished data).

**Figure 2:**
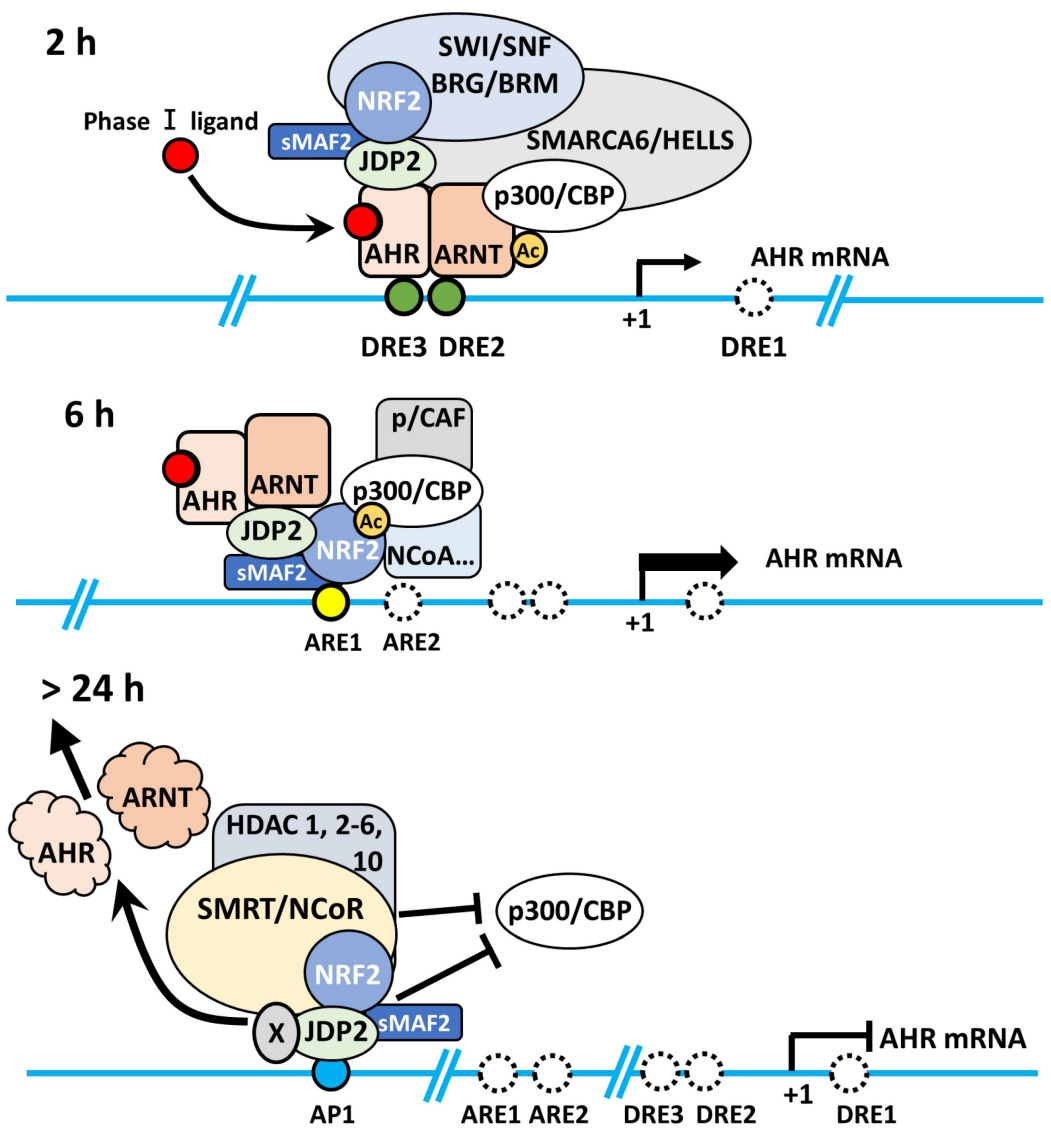
** Modes of *AHR* promoter activation in a spatiotemporal manner.** Schematic representation of TCDD-induced *AHR* activation through the AHR-JDP2, NRF2-JDP2, and AHR-NRF2 complexes to increase ROS production, cell spreading, and apoptosis in wild-type MEFs. In *Jdp2*^-/-^ MEFs, only a residual amount of AHR-ARNT is recruited to the DRE2 and DRE3 elements of the *AHR* promoter. Recruitment to DRE occurs at DRE2 and DRE3 after a 2-h exposure to TCDD. After 6-h exposure, this complex moves to ARE1 and ARE2 because AHR degradation starts via ubiquitin complex activity. After 24 h, the AHR activity is due to JDP2 binding to the AP-1 site in the *AHR* promoter. This TCDD-induced *AHR* promoter activation appears to be performed by the AHR-NRF2-JDP2 battery, as previously described 21.

Subsequently, the NRF2-JDP2 in the complex can associate with AREs at 6-18 h as a mode of spatial regulation. This time- and space-dependent regulation of the AHR-NRF2-JDP2 complex was due to its binding preference first for DRE2/3 (AHR in the complex binds to DRE first) and later for ARE1 (NRF2 in the complex subsequently binds to ARE). This type of sequential and spatial selection occurred by the interaction of phase I ligand with AHR in this complex because exposure to phase II ligands did not stimulate the *AHR* promoter at 2-6 h 21. This key spatiotemporal regulation initially might be performed by the chromatin remodeling activities of ligand-bound AHR and histone chaperone JDP2, because JDP2 deletion did not stimulate *AHR* promoter activation even at 2-6 h after binding 21. Thus, JDP2 might affect the order of *cis*-element binding via its histone chaperone function. Moreover, initial binding of the AHR-NRF2-JDP2 complex to the DRE was determined by chromatin opening facilitated by JDP2-brahma-related gene 1 (BRG1) or JDP2-cohesin or the TCDD bound AHR-ARNT with CBP/p300 acetylase, leading to subsequent recruitment of the complex to the DRE2/3 (**Fig. 2**).

Later, the AHR-NRF2-JDP2 complex was directed to the AREs at 6-18 h. Subsequently, the degradation of nuclear AHR by AHR degradation machinery might start at 18-24 h gradually because AHR was not detected in the nucleus at this time point 21. Thus, the NRF2-JDP2 complex appeared to predominantly mediate ARE-dependent recruitment. Indeed, the expression of *AHR* promoter-luciferase and reactive oxygen species (ROS) production gradually decreased after 6-24 h 21. AHR promoter activity, which initially depended on various factors, became dependent on the AP-1 element after 24 h. In addition, JDP2 on AP-1 site might recruit the HDAC complex to induce the histone deacetylation, and INHAT induced by JDP2 to close the chromatin 36,37. Concurrently, the overall *AHR* promoter activity itself began to decline gradually after this point 21.

Ubiquitin-related events also regulated the degradation of AHR. Ubiquitin-like with prolyl hydroxylase domain and RING finger domains 1 (UHRF1) is a multidomain protein originally defined as being involved in the maintenance of DNA methylation. It was found to bind hemimethylated DNA and recruit DNMT1 to the DNA replication foci 106. Furthermore, UHRF1/DNMT1 was involved in the hypermethylation of promoters in tumor suppressor genes to downregulate their expression and inhibit cellular apoptosis 107. Moreover, UHRF1 acetylated by Tat-interactive protein-60 inhibited colon cancer cell growth through the re-expression of JDP2 108.

Jdp2 was also involved in antioxidation function with Nrf2-MafK complex by suppressing ROS generation and increasing *ARE* response gene promoter activity after long-term exposure of 12-*O*-tetradecanoylphorbol-13-acetate (TPA) 109. Thus, at this stage, JDP2 played a critical role in suppressing the AHR response by NRF2 dependent anti-ROS reaction to maintain the ROS homeostasis.

As describe above, the spatiotemporal regulation of the *AHR* promoter by the AHR-NRF2-JDP2 complex was supported by the following evidence using wild-type MEFs 21,23. (i) This dogma was verified using ChIP and co-immunoprecipitation/western blotting of AHR, NRF2, and JDP2 in the nuclear and cytoplasmic fractions, which was time-dependent after TCDD exposure, and by mutations of the DRE2/3, ARE1, and AP-1 sites in the *AHR* promoter to demonstrate the time- and space-dependent activation of AHR-luciferase 21 (**Fig. 1**). (ii) Preliminary studies were performed using JDP2 mutants in which amino acids that interacted with either AHR or NRF2 were mutated. We identified FL34R zipper region (amino acid positions 114 and 121) and N91A basic region (amino acid position 91) mutants of JDP2, in which the respective *AHR* promoter and *NRF2* promoter luciferase activities were lost 21,36,37. Regarding the JDP2/AHR signaling pathway, JDP2 loss inhibited cytoskeletal remodeling, cell spreading, and cell migration 21.

### 5.2. Epigenetic chromatin regulation of AHR and its target gene products

Regarding the role of JDP2 chaperone in the basic RNA transcription machinery, JDP2 might promote chromatin-stimulating histone modifications to recruit the RNA Pol II transcription initiation complex to the *AHR* promoter with phase I complex AHR-ARNT and phase II complex NRF2-sMAF bound to CBP/p300 HAT, HDAC family/bromodomain-containing 4, chromatin remodelers, such as SWI/SNF complex members and mediators, which are associated with the Pol II general transcription factors 110-112, and transcription elongation factor b complex (cyclin-dependent kinase 9/cyclin T1) transcriptional machinery 113,114. BRG1 plays a role in chromatin accessibility, Pol II complex binding, and nascent RNA generation by controlling nucleosome positioning 115.

Tumor suppressor gene products can suppress *AHR* promoter activity. TCDD exposure induced the promoter methylation of the tumor suppressor genes p16^INK4a^ and p53, and subsequently repressed their transcription in keratinocytes 66, indicating that the unmodified sequences of DRE as AHR binding sites are important for DNA binding by the ligand-bound AHR/ARNT complex.

DNA methylation alterations at the loci cg14647125 and cg23916896 (both located in the AHR repressor gene body) are linked to ulcerative colitis risk (*P* = 0.001 and 0.002, respectively). The biological pathways underlying the effects of smoking on the pathogenesis of inflammatory bowel disease, potentially involving the AHR repressor, have been identified 116,117.

The available miRNA databases miRTarBase 8.0 to 9.0 (06/27/2024; analyzed using miRNet 2.0 118 and miEAA 2023 119,120) showed that almost 100 miRNAs are potentially involved in the posttranscriptional regulation of AHR. Here, we did not focus on the miRNA regulation of *AHR*. Furthermore, the specific mechanism and extent of the link between AHR and epigenetics warrant further investigation.

### 5.3. JDP2 functions as a histone chaperone in chromatin regulation

JDP2 functions as a histone chaperone, HAT inhibitor for CBP/p300 36, and a recruiter of HDACs (such as 1-6 and 10) for inhibiting histone acetylation 114,121,122. JDP2 bound to the reconstituted chromatin and intact chromatin in vitro and showed chromatin assembly. JDP2 also bound core histones directly through its histone-binding region (amino acids 35 to 70), which was distinct from its basic zipper region 36. It also inhibited the p300-induced histone acetylation on H3 and H4 (specifically H4K8 and H4K16), via its inhibitor of HAT (INHAT) activity domain (amino acids 35 to 102) 36.

This coordinated action might be possible through direct protein-protein interactions of JDP2 with AHR or NRF2 because possibly, it has different regions that bind to AHR and NRF2. In addition, the knockdown experiments of AHR, ARNT, NRF2, and JDP2 showed significantly reduced *AHR* promoter activity, and the addition of JDP2 in *Jdp2^-/-^* MEFs could rescue the *AHR* promoter activity 21,23. Thus, the players of the AHR-NRF2-JDP2 axis can interact with each other in a time- and space-dependent manner to bind the DRE2/3, ARE1, and AP-1 sites in the *AHR* promoter. Thus, JDP2 might function as a histone chaperone in DRE and ARE cis-element mediated *AHR* expression. AHR, NRF2, and JDP2 enhanced the *AHR* transcription activity in a synchronized manner, which was confirmed using studies involving mutants of each cis-element in the *AHR* promoter and ChIP assay 21,23,36. Thus, JDP2 might regulate the recruitment of CBP/p300 and HDACs, which were involved in chromatin remodeling to mediate the open-close chromatin transition during the transcription of *AHR*.

Furthermore, JDP2 is involved in multiple processes/functions, including cell growth, cellular senescence, cell death, tumor control and enhancement, stemness, and pluripotent capacity 36. JDP2 downregulated p53 transcription and promoted tumorigenesis in p53 heterozygous conditions. JDP2 also inhibited ultraviolet-induced apoptosis by reduced expression of p53 123 and by oncogenic transformation 124 or tumor suppression in a cell type-specific manner 125. Conversely, Price et al. showed that JDP2 was responsible for increasing p53 transcription by decreasing the expression of murine double minute 2 protein in human H1299 non-small cell lung cancer (NSCLC) and MCF7 breast cancer cell lines, which mutated Ha-Ras/K-Ras and PI3K/AKT signaling, respectively 126. However, in some cases, JDP2 was implicated in leukemogenesis and exhibited oncogenic potential. Transposon-mediated insertions could lead to JDP2 upregulation, while simultaneously causing the downregulation of tumor protein p53 (Trp53), a tumor suppressor gene 127. In patients with T-cell ALL (T-ALL), JDP2 promoted cell survival by upregulating anti-apoptotic myeloid cell leukemia-1 (MCL1) protein. The overexpression of JDP2 led to MCL1 upregulation and steroid resistance *in vivo*, which may contribute to the poor survival rates observed in patients with T-ALL 128.

Moreover, JDP2 mediated cell cycle arrest through cyclin A2 129. JDP2-mediated growth suppression was inhibited by downregulating both p16^Ink4a^ and adenosine diphosphate-ribosylation factor (Arf or p14Arf). Conversely, forced expression of p16^Ink4a^ or Arf led to a decrease in the proliferation of *Jdp2^-/-^* MEFs. Thus, JDP2 induced p16^Ink4a^ and Arf during stress conditions, resulting in cell cycle arrest through both the p16^Ink4a^/RB and Arf/p53 pathways via alteration of H3K27 methylation 130,131. Therefore, JDP2 played a critical role in Ink4-dependent ROS regulation and senescence through the AHR‒NRF2 cascade by modulating polycomb and trithorax proteins.

As described above, JDP2 plays a role in both chromatin remodeling and HAT inhibition 36, whereas activating transcription factor 2 (ATF2), as a partner of JDP2, has intrinsic HAT or enhanced HAT activity 132-135. JDP2 suppressed ATF2 function through HDACs 121,136. CBP/p300 could acetylate NRF2 67,68, which enhanced the ARE response by increasing the DNA-binding activity of NRF2 and promoted the upregulation of ARE-regulated genes through its interactions with ARF proteins, such as p14^Arf^ (p19^Arf^ in mouse) 137.

**Figure 3:**
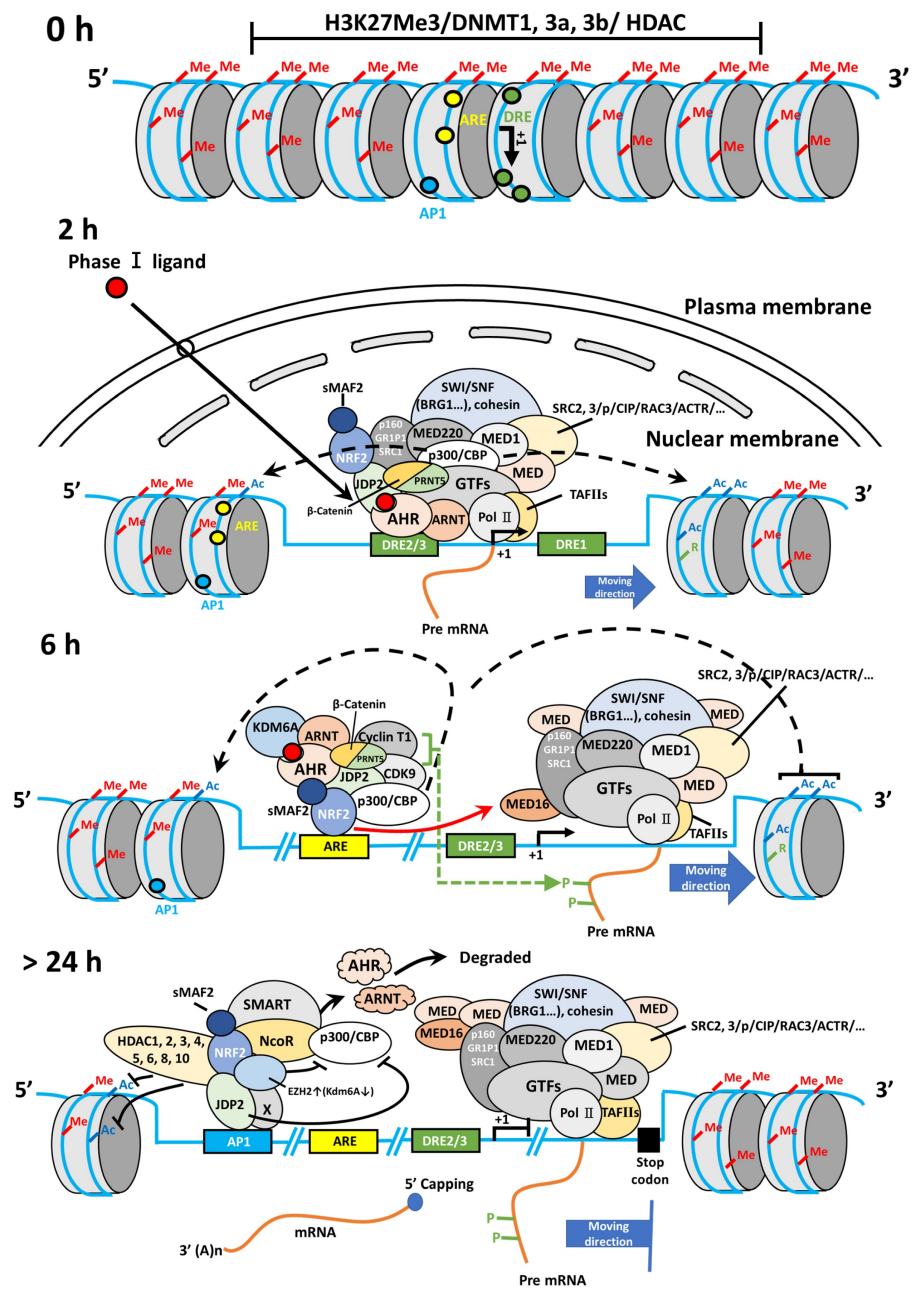
** Hypothetical modeling of the AHR-NRF2-JDP2 axis**. Chromatin remodeling and epigenetic regulation of the *AHR* locus were involved in the function of histone acetylation and deacetylation of the AHR-NRF2 complex and the histone chaperone JDP2. In TCDD-induced MEFs, TCDD-bound AHR enters the nucleus and binds to ARNT within 2 h of exposure to TCDD. Then, AHR-ARNT recruits NRF2/sMAF and JDP2, which interact with SWI/SNF complexes, such as BRG1 and cohesin SM3, which in turn open the closed chromatin. Subsequently, AHR-ARNT with coactivator CBP/p300 binds to DRE2/3 of the *AHR* promoter. Then, RNA polymerase complexes are recruited to the transcription start site. After 6-h exposure to TCDD, the AHR-ARNT complex moves to the ARE1 site through the NRF2-sMAF complex, and recruits coactivator complexes, such as p160/SRE1/2/NCOA, pCIP/AIB/ACTR, and CBP/p300, with Pol II, mediator complex including MED16 112, and cyclin-dependent kinase 9/cyclin T1, to mediate mRNA elongation with C-terminal domain phosphorylation in cooperation with positive transcription elongation factor b 113. After 24-h exposure, nuclear AHR is degraded, and the AP-1 site remains active for *AHR* transcription to maintain the coactivator complex. After greater than 24-h exposure to TCDD, JDP2 at the AP-1 site can recruit corepressors, such as nuclear receptor corepressor/silencing mediator for retinoic acid and thyroid hormone receptors and HDACs 1-6 and 10 and inhibit the histone demethylase activity mediated by lysine demethylase 6A and the coactivator CBP/p300 to terminate *AHR* RNA transcription and close the chromatin at the *AHR* locus. This Figure was published in Biochemical Pharmacology, Vol. 233, Wuputra K, Hsu WH, Ku CC, Yang YH, Kuo KK, Yu FJ, Yu HS, Nagata K, Wu DC, Kuo CH, Yokoyama KK, The AHR-NRF2-JDP2 gene battery: Ligand-induced AHR transcriptional activation., 116761, Copyright Elsevier B.V., 2025, and we were permitted to reuse and modify from Elsevier B.V.

To identify the JDP2 function at the promoters of *AHR* and *NRF2*, a genome-wide ChIP study of the transcriptional activation domain should be conducted. The critical residues of JDP2 that interact with CBP/p300, CBP/p300-associated factor (pCAF), ATF2, Tat-interactive protein-60, ARF, p16^Ink4a^, and cohesion (or condensing) should be investigated using capture Hi-C, 3C, 4C, and 5C assays. Other acetylated histone groups of histones H3 and H4 should also be assessed to identify JDP2's regulatory functions. These investigations might help elucidate the molecular mechanisms of the AHR-NRF2-JDP2 axis (**Fig. 3**).

The AHR-NRF2 gene battery was first demonstrated in keratinocytes 26-32. Subsequently, this concept was further explored using MEFs 21. This autoregulation of the *AHR* promoter activation was also observed for other phase I enzyme ligands besides TCDD, including formylindolo[3,2-*b*]carbazole, BaP, and tryptophan metabolite Kyn, in wild-type MEFs.

## 6. Pathological significance of the AHR-NRF2-JDP2 axis at the organismal level

### 6.1. KO or knockdown of the AHR, NRF2, and JDP2 pathways in mice

The pathological significance of the AHR-NRF2-JDP2 axis at the organismal level is the key issue to link this gene battery to the development of therapeutics for clinical or preclinical application. However, detailed studies on double KO (DKO) or triple mice of Ahr-Nrf2, Ahr-Jdp2, and Jdp2-Nrf2 have not been reported. Ahr-deficient mice are viable but do not respond to phase I enzyme ligands. These mice have a reduced liver weight (reduced by 75%) and delayed hematopoiesis ability and hepatic microvascular steatosis 138. Although Ahr-deficient mice do not generate spontaneous tumors 139,140, several studies indicated that AHR functions as a tumor suppressor in a context-dependent manner.

Shin et al. 25 reported that NRF2-regulated AHR signaling affects xenobiotic metabolism, via the CYP450 family, and adipogenesis. Yamamoto's group 141 reported that Ahr-Nrf2 DKO mice were viable and fertile and had no apparent phenotypic alterations. They postulated that the NRF2 pathway affected AHR-dependent pathways such as apoptosis and development. However, there have been no additional reports using Ahr-Nrf2 DKO mice. Nrf2-KO mice did not exhibit any obvious phenotype 142, except for discolored teeth due to iron transport defects 143.

JDP2 is a transcription factor with histone chaperone activity, which regulates the chromatin structure of the AP-1/ATF loci 21,36,37,144. It repressed cell proliferation and regulated the cell cycle by targeting cyclin A 129. In addition, JDP2 enhanced reprogramming potency in MEFs and could replace octamer-binding transcription factor 4 (OCT4) among the Yamanaka reprogramming factors. JDP2 has been shown to anchor five non-Yamanaka factors, including inhibitor of DNA binding 1, Jumonji C histone demethylase 1B, liver receptor homolog-1, Spalt-like transcription factor 4, and Glis family zinc finger 1, to reprogram MEFs into induced pluripotent stem cells (iPSCs) 145. JDP2 and OCT4 reprogram cancer cells into iPSC-like cells 146,147. *Jdp2* KO mice were small and had short tail but exhibited no other obvious phenotype (Yokoyama unpublished data). JDP2 plays a key role in bone homeostasis and host defense by regulating osteoclast and neutrophil differentiation 148. *Ahr-Jdp*2 DKO mice are embryonic lethal (unpublished data); however, knockdown of Jdp2 in *Ahr* KO mice has been used to demonstrate enhanced tumorigenesis of LSL-kRAS^G12D^p53^lox/lox^ pancreatic adenocarcinoma 21. Thus, JDP2 is the upstream gene of AHR. Conditional KO or knockdown mice should be generated for further assessment of the AHR-NRF2-JDP2 gene battery as described below.

Studies using conditional KO or knock-in mice targeting the skin or related cells have demonstrated that AHR-ARNT and the NRF2/Keap1 pathway play a crucial role in regulating the skin barrier and epidermal barrier function. Ahr^flox^::K14-Cre mice demonstrated increased trans-epidermal water loss after tape stripping in the upper layers of the stratum corneum, indicating that AHR plays a role in maintaining skin barrier function 149. In the case of the AHR-interacting partner ARNT, these mice showed an impaired epidermal barrier, increased trans-epidermal water loss, severe dehydration, and body weight loss. They died within 24 h after birth 150,151. Transgenic mice with a constitutively active Nrf2 mutant (caNrf2) gene in keratinocytes showed scaling and dry skin 152. The caNrf2 (lacking NehN2 domain)::K5-Cre mice showed epithelium thickening (acanthosis) and severe hyperkeratosis in the skin 153. Loricrin (Lor) is a structural protein in the cornified cell envelope present on the surface of terminally differentiated epidermal cells, which is composed of a complex network of cross-linked proteins, primarily held together by disulfide/ε-(γ-glutamyl) lysine cross-linkages. In mice where NRF2 activity was inhibited (Lor-KO::dnNrf2 mice), a critical skin barrier component was affected, leading to severe barrier dysfunction and death within 24 h 154.

The crosstalk between AHR and NRF2 also plays a role in immune and inflammatory responses. The forced expression of NRF2 caused the upregulation of IL-17A and IL-22 in CD4^+^ T cells polarized to Th17 cells in Nrf*2^-/-^* and *Ahr^CD4^* KO mice. However, the IL-22 response in CD4^+^ T cells, not IL-17A, was regulated by NRF2 via the AHR pathway. Specifically, NRF2 activation promoted IL-22 production in CD4^+^ T cells in an AHR-dependent manner 155. Foxn1-Cre-induced Ahr KO (*Ahr* KO) mice exhibited a significant reduction in the regenerative ability of thymus cells. For example, the Ahr agonist 6-formylindolo [3,2-*b*] carbazole and AHR inhibitor CH-223191 accelerated and blocked regeneration of the mouse thymus, respectively, and this could not be reversed by the introduction of exogenous IL-22. *Ahr* KO mice exhibited a decreased IL-22 receptor alpha 1 (IL-22RA1) expression. Thus, both AHR and IL-22RA1 were critical for thymus regeneration and implicated in the pathogenesis of chronic graft-versus-host disease 156.

Experiments involving colitis *in vivo* in mice or *in vitro* colon organoid models were performed to determine how the expression of mucin 2 protein was altered with or without AHR in intestinal epithelial cells (IECs) in response to indole-3-carbinol. On comparing wild-type mice to IEC-specific Ahr KO mice (Ahr^ΔIEC^), AHR expression was found to be essential in IECs for indole-3-carbinol-mediated protection during colitis. The loss of AHR impaired the expression of mucin protein 2 independently of IL-22 157.

### 6.2. Tumor suppression of AHR-p53 in cancer

Next, we were interested in determining whether one or both alleles of Trp53 can affect tumorigenesis. The p53 transcription factor is a multifunctional protein with key roles in regulating the cell cycle, apoptosis, senescence, reprogramming, cell migration, and genome maintenance 158. Homozygous mutations in the p53 gene were detected in approximately 50%-60% of human cancers, of which 90% were missense mutations in approximately 190 different codons localized in the DNA-binding region 158-167. Inheritance of the p53 mutations was the primary cause of Li-Fraumeni syndrome, which significantly increases the risk of cancer 161. In cancer, mutations in one p53 allele were frequently accompanied by the deletion or inactivating mutations in the remaining p53 allele 162.

The role of AHR signaling in tumorigenesis in the case of p53 loss has not yet been established. Thus, the lifespan and tumor spectrum of Ahr-depleted mice in p53 heterozygous and p53 KO backgrounds were assessed 163-167. Ahr and p53 DKO mice had a short lifespan with reduced embryo survival and developed tumorigenesis compared with control p53 null mice. Taken together, the findings showed that AHR functions as a tumor suppressor in p53-depleted mice; thus, developing anticancer drugs that promote this tumor-suppressive activity is a promising therapeutic strategy 165. Ahr*-*depleted mice developed more aggressive tumors than their wild-type counterparts in the transgenic adenocarcinoma of the mouse prostate model 166 and showed increased liver tumors induced by diethylnitrosamine in male mice compared with their wild-type AHR littermates 167.

### 6.3. Dual role of AHR in oncogenic and tumor suppressor functions

IECs-specific knockdown of Ahr led to the expansion of clonogenic progenitor cells in mice with mutations in adenomatous polyposis coli (APC) and Kras genes (*Apc^S580/^*^+^;* Kras^G12D/^*^+^) and promoted cell growth in the gut epithelium to increase cecum and colon cancer in mice 168. Intestinal-specific *Ahr* KO mice showed increased basal stem cells and crypt injury-induced cell growth in a colitis-associated tumor model 169. Moreover, Ahr suppressed intestinal tumorigenesis in APC^Min/+^ mice 170 and high AHR expression was associated with improved patient survival in some cancers, indicating that Ahr can be targeted for the inhibition of cancer cell proliferation 171-175. In other multiple cancer models, Ahr deletion promoted increased tumorigenesis, but the precise genetic and molecular mechanisms remain unclear 176.

Ahr linked to wingless-related integration site (Wnt)/β-catenin signaling played a critical role in tumor suppression, particularly in intestinal and liver cancers. AHR loss, coupled with Wnt/β-catenin signaling activation, was speculated to promote tumorigenesis in cancer models. This hypothesis is supported by studies in models where AHR was deleted or suppressed, resulting in increased Wnt activity and enhanced tumor development. Specifically, mutations in APC and AhR deletion have been observed in Wnt/β-catenin-driven cancer models 172-175,177-179.

In some cancers, such as colon cancer, AHR had dual roles in tumor oncogenesis and tumor suppression by promoting the integrity of the epithelial barrier, inhibiting inflammation, and antagonizing signals downstream of Wnt/β-catenin during the regenerative process. AHR restricted the proliferation of stem cells by inhibiting the expression of OCT4, SOX2, c-Myc, and NANOG factors 180, and AHR activation could increase the differentiation capacity in multiple cancer types 181. Furthermore, AHR could antagonize oncogenic signaling, such as PI3K/AKT-dependent growth factor 182, sonic hedgehog, and transforming growth factor-β signaling 183. AHR was normally enriched on several oncogenic genes, such as those in the transforming growth factor-β and NRF2 signaling pathways 184. Therefore, AHR functioned as a tumor suppressor or an oncogene in a cell type-specific manner or depending on the status of p53 mutation or deletion, or p16^Ink4a^ methylation.

### 6.4. AHR-NRF2 in gut microbiota

Polycyclic aromatic hydrocarbons (PAHs) induced carcinogenesis by activating AHR in gut microbiota, which metabolized PAHs to highly reactive carcinogenic intermediate compounds 185. The gut microbiome secreted many metabolites in the tumor microenvironment, such as short-chain fatty acids (SCFAs), formate, and tryptophan-derived indoles, which promoted immune tolerance and metastasis via AHR signaling. For example, the production of TNF-α and IL-6 in tumor-associated macrophages and dendritic cells was observed in response to lipopolysaccharide (LPS). Indoleamine 2,3-dioxygenase (IDO) activity was stimulated by LPS in resident antigen-presenting cells and tumor cells, leading to the increased production of Kyn from tryptophan, which activated AHR and subsequently led to increased immune tolerance.

AHR was found to play a crucial role in microbe-mediated oncogenesis as a sensor molecule for several microbial metabolites in the gut. Because most studies have investigated *Fusobacterium nucleatum*, additional studies are needed to understand fully the possible cross talk between AHR and other bacterial species in colorectal cancer, such as *Staphylococcus gallolyticus*, *Bacteroides fragilis, Escherichia coli B2*, *Enterococcus faecalis*, and *Peptostreptococcus anaerobius.* Furthermore, the role of microbiota in stimulating immune responses and modulating responsiveness to immunotherapy, including via AHR signals, required further examination 185.

The AHR/NRF2 pathway was activated in the colon as described above, whereas the nucleotide-binding oligomerization domain (NOD)-like receptor family pyrin domain containing 3 pathway was downregulated. Indole-3-lactic acid, which was an AHR ligand produced by *Bifidobacterium bifidum* FL-276.1 and FL-228.1, regulated the AHR/NRF2/NOD-like receptor family pyrin domain containing 3 pathway in Caco-2 cells to upregulate the tight junction proteins and protected the integrity of the epithelial barrier. Such studies were conducive to promoting clinical trials and developing probiotics for alleviating colitis 182.

*Lactobacillus rhamnosus* GG (LGG)-derived exosome-like nanoparticles (LDNPs) were released by the probiotic LGG, activating the AHR-NRF2 axis in the intestine, which can be blocked using LDNP inhibitors. The LDNPs were found to protect intestinal barrier function. These nanoparticles also protected against experimental alcohol-associated liver disease via intestinal AHR/IL22/Reg 3-related and NRF2 signaling pathways, leading to decreased bacterial translocation and LPS release 186.

### 6.5. AHR/NRF2 in the gut-liver axis

Hepatic sinusoidal obstruction syndrome (HSOS) was a well-known serious syndrome that can arise after autologous and allogeneic hematopoietic stem cell transplantation, and during treatment of certain cancers, such as Wilms tumor, rhabdomyosarcoma, and ALL. Replenishing glutathione with N-acetyl cysteine may be a reasonable approach to decreasing the risk of HSOS after cytotoxic therapy and myeloablation, but it may also decrease the efficacy of the chemotherapy for malignancies. Lower levels of tryptophan were produced and AHR stimulation was significantly reduced in the rat HSOS model. However, when injured HSOS rats were exposed to AHR ligands, the liver phenotype recovered by activation of AHR and NRF2 pathways in the liver 187.

In a mouse hepatic steatosis model, treatment with sulforaphane (SFN), which was an NRF2 agonist, reversed the steatosis by NRF2 activation. Thus, SFN treatment during a high-fat diet modulated lipid metabolism via the AHR-sterol regulatory element-binding protein 1 pathway by changing the gut microbiota, leading to the conversion of tryptophan to indole-3-acetic acid, which was a potent ligand for AHR 188. Lansoprazole, which was a drug for treating gastric ulcers, activated the antioxidant stress response in rat hepatocytes, potentially treating oxidative hepatic damage via cross talk between AHR and NRF2 189.

In addition, the carotenoid lycopene can act as an antioxidant drug to inhibit oxidative stress by modulating the AHR-NRF2 axis in the liver 190. In addition, *S*-allylmercaptocysteine, which was an antioxidant drug, ameliorated metabolic dysfunction-associated steatotic liver disease by modulating the AHR-NRF2 axis in the liver. This drug targeted antioxidation-related genes, such as NQO-1, and potentially inhibited the inflammasome of NOD-like receptor protein 3/6 191.

Many studies have shown that AHR-NRF2 cross talk occurs in the gut, liver, and gut-liver axis 192,193. This study encompassed various pathologies that were involved in the AHR-NRF2 axis. This cascade may provide valuable insights into future preclinical therapy. New ongoing clinical trials are investigating the potential of food compounds that interact with NRF2 or AHR pathways in inflammatory diseases. Curcumin has been studied for its potential benefits in treating patients with chronic kidney disease (CKD). The findings confirmed the anti-inflammatory properties of curcumin, which acted via the NRF2 axis 194.

However, more precise investigations are needed regarding the AHR-NRF2 cross talk in the gut or liver. Recently, it was shown that quercetin could improve gut barrier function in dextran sulfate sodium-induced colitis (ulcerative colitis) by regulating neutrophil extracellular traps and it could activate AHR and subsequently upregulate ARNT in neutrophils to regulate these extracellular traps 195.

The phase I enzyme ligand TCDD can induce expression of the phase II enzyme pyruvate kinase muscle isoform 2 (PKM2) in normal differentiated hepatocytes. PKM2 was a key enzyme in aerobic glycolysis, which contributed to cancer cell metabolism. The cooperative regulation between NRF2 and AHR inducing PKM2 was assessed in mice treated with TCDD. Approximately 579 genes among 842 NRF2-enriched regions showed both NRF2 and AHR enrichment. Sequence analysis of regions showed overlapping NRF2 and AHR enrichment in the respective ARE or DRE sites. Although 18 regions possessed both motifs, which were responsible for either AHR or NRF2 signaling, NRF2 showed negligible enrichment within a closed PKM2 chromatin region, whereas AHR was enriched 29-fold. In addition, TCDD activated PKM2 in primary hepatocytes from wild-type and NRF2-deleted mice. Although both NRF2 and AHR can cooperate to regulate antioxidant gene expression, the induction of PKM2 by TCDD was independent of NRF2 activation 196. PKM2 was a coactivator for *AHR* 194 and *PKM2* promoter was found to contain DRE sites to which AHR could bind.

### 6.6. AHR-NRF2 in the skin

Atopic dermatitis (AD) is a chronic inflammatory skin disorder characterized by extensive skin barrier dysfunction and increased expressions of IL-4 and IL-13. The barrier dysfunction of AD correlated with the downregulation of barrier-related molecules such as filaggrin, Lor, and involucrin. Natural or medicinal ligands for AHR were considered potent upregulators of filaggrin, Lor, and involucrin. IL-4, IL-13, IL-22, and IL-17A can induce oxidative stress; hence, antioxidative AHR agonists, such as coal tar, glyteer, and tapinarof showed therapeutic efficacy for AD 197,198.

### 6.7. AHR-NRF2 in the lung

The PAH-AHR signaling pathway was a critical axis in promoting lung inflammation and impairing lung function in many lung diseases 199. The levels of hydroxynaphthalene, hydroxyphenanthrene, and hydroxyl PAHs were significantly elevated in the urine of patients with lung cancer compared with healthy controls 200. Activation of the PAH-AHR pathway promoted systemic inflammation and exacerbated the progression of lung diseases, such as chronic obstructive pulmonary disease and lung cancer.

In the lung-gut axis, PAH exposure induced intestinal flora dysbiosis, leading to impaired intestinal barrier function and increased inflammation. As a therapeutic strategy, diet-derived AHR ligands, probiotics, and SCFAs may ameliorate PAH-mediated chronic inflammation and lung disease. Thus, the regulation of inflammation and intestinal dysfunction mediated by AhR signaling can inhibit systemic inflammation in patients with inflammatory lung diseases 201.

Regarding the AHR-NRF2 axis in lung disease or injury, hyperoxia (>95% O_2_) led to the induction of CYP1A1, NQO1, and GSTs 202-204. By contrast, the hyperoxia-induced CYP1A2 upregulation did not involve AHR signaling 204. AHR loss increased ROS generation in fetal primary lung cells in response to hyperoxia and resulted in higher susceptibility to hyperoxia lung injury in adult and newborn mice. Wang et al. demonstrated that *Cyp1a2* KO (which is predominantly expressed in the liver) increased susceptibility for hyperoxia lung injury. Thus, the Cyp1a2-mediated metabolism of F_2_-isoprostanes PGF2α, might be the target for protection against hypertoxic lung injury 205.

Another possibility is the AHR-NF-κB-RelB interaction. AHR was shown to interact with RelB and modulate its expression 206,207. AhR-deficient fetal human pulmonary microvascular endothelial cells showed higher hyperoxia-induced ROS generation, cleavage of poly (adenine dinucleotide phosphate-ribose) polymerase, and cell death than AhR-sufficient fetal human pulmonary microvascular endothelial cells 202. The expression of CYP1A1, NQO1, SOD1, and nuclear RelB decreased in AHR-deficient cells. These findings supported the hypothesis that decreased antioxidant enzymes and RelB activation in AhR-deficient cells were associated with increased hyperoxic injury compared with AhR-sufficient cells. RelB acted as a negative regulator of the proinflammatory NF-κB pathway, possibly by its interaction with p50, thereby reducing the amount of p50 to form active dimers with p65 in the NF-κB complex 208. 3,3′-Diindolylmethane (DIM), which was an active phytochemical derivative, induced ferroptosis in NSCLC cells. This treatment resulted in increased cellular Fe^2+^, ROS, and malondialdehyde levels; decreased cellular glutathione, AHR, NRF2, and glutathione peroxidase 4 (GPX4), and inhibition of the mitochondrial membrane potential. These findings provided useful knowledge on DIM treatment and clinical research in patients with NSCLC 209. The effects of DIM-induced ferroptosis can be reversed using the AHR receptor antagonist CH-223191, ferroptosis inhibitor Fer-1, and ROS scavenger NAC. Overexpression of NRF2 reversed DIM-induced ferroptosis. Thus, DIM induced cancer cell ferroptosis through the AHR/NRF2/GPX4 axis.

## 7. Association of JDP2 with AHR-NRF2

The function of JDP2 with AHR-NRF2 at the organismal level has not yet been reported. The oxidation and antioxidation stresses including metabolic stress, replication stress to control the oxygen, ATP, NAD(H), NADP(H) or peroxides might be possible to maintain inflammation, allergy, aging, disease, or cancers. The following functions of JDP2 might be involved in regulating the AHR-NRF2 gene battery to regulate the ROS balance: (i) regulation of expression of solute carrier family 7 member 11 (SLC7A11) through the AHR-NRF2 axis to regulate ferroptosis and cell death; (ii) control of cardiac remodeling and function; (iii) control of oncogenicity in T-cell lymphoma, which can lead to the development of cancer; (iv) control of *in vivo* bone homeostasis and host defense by regulating neutrophil differentiation; and (v) chromatin remodeling and epigenetic regulation of AHR, NRF2, and JDP2.

IDO1 is a key enzyme of tryptophan catabolism in the Kyn pathway. IDO1 activation inhibited ferroptosis in erastin-exposed lung cancer cells and decreased lipid peroxidation and ROS production 206. IDO1 stimulated NRF2 expression through activation of the AHR axis. It also upregulated the expression of the SLC7A11 ion channel, enhanced the pentose phosphate pathway via the AHR-NRF2 axis, and led to decreased generation of nicotinamide adenine dinucleotide phosphate and glutathione, thereby inhibiting ferroptosis. Furthermore, *trans*-3-indoleacrylic acid, which was a metabolite produced by *P. anaerobius*, promoted colorectal carcinogenesis by inhibiting ferroptosis independently of the enzyme GPX4. Instead, it mediated this action through the AHR/aldehyde dehydrogenase 1 family member A3/ferroptosis suppressor protein 1/coenzyme Q10 pathway 207. In fact, JDP2 regulated ROS production and glutathione levels through SLC7A11 expression in granule cell progenitors 210,211. In addition, JDP2 induced the GABR6 subpopulation of mouse granule cell progenitors to differentiate into Purkinje cells 212.

Indole-2-lactic acid (ILA) as a gut microbiota metabolite was found to play a role in mitigating doxorubicin-induced cardiotoxicity (DIC). It is a ligand for AHR, which activates the NRF2 signaling pathway through the AHR-NRF2 axis. The inhibitory function of ILA against ferroptosis was abrogated by AHR loss. In addition, the beneficial effects of ILA on DIC were eliminated in Nrf2-deficient mice. Thus, ILA exerted therapeutic functions against DIC by blocking ferroptosis via activation of the AHR-NRF2 axis 213. The uremic toxin indoxyl sulfate induced cardiac fibroblast activation and cardiac fibrosis in CKD. It also induced the proinflammation of neonatal mouse cardiac fibroblasts partly via the AHR pathway 214. Thus, targeting AHR is a strategy to mitigate vascular inflammation and reduce the cardiovascular burden in CKD 215.

JDP2 played a role in the pathology of myocardial hypertrophy. Jdp2/activating transcription factor 3 (Atf3) DKO mice showed resistance to maladaptive cardiac remodeling processes and exhibited preserved cardiac function. The expression of both ATF3 and JDP2 was important for cardiac function in healthy and diseased hearts 216,217.

Inorganic arsenic shows cytotoxicity in human lymphoblastoid cells. The NRF2/Keap1 pathway was not the only cascade that functioned in response to acute doses of arsenic in lymphoblastoid cells. Other phase II enzymes (e.g., heme oxygenase 1) regulated by NRF2 can function as both acute and chronic biomarkers of arsenic exposure 218.

JDP2 was abnormally expressed in the T-ALL subset and associated with poor survival. It was required for T-ALL cell survival because its deletion led to apoptosis. Mechanistically, JDP2 controlled prosurvival signaling through direct transcriptional regulation of the anti-apoptotic protein MCL1 127,128,219.

Treating fine particulate matter with a diameter of ≤2.5 μm (PM2.5) with a strong acid at a high temperature hydrolyzed any protein content and removed trace elements. This reaction of PM2.5 with a strong acid at a high temperature terminated the AHR-dependent pathway, decreasing the eosinophil numbers in bronchoalveolar lavage fluid cells, lowering IL-13 and CXCL3, and reducing the peribranchial inflammation. By contrast, neutrophil numbers in bronchoalveolar lavage fluid cells and levels of macrophage inflammatory protein 2 alpha, epidermal growth factor receptor, NRF2, Toll-like receptor 4, and 4-hydroxy-2-nonenal in the lung were increased. PM2.5-bound proteins and acid-soluble metals might underlie the pathogenesis of PM2.5-induced allergic airway inflammation 220. In addition, diesel exhaust exposure induced neutrophilia and lymphocytosis in humans. These responses were linked to the activation of key intracellular signaling pathways, including NF-κB, c-Jun, and mitogen-activated protein kinases, and the increased production of inflammatory mediators. Diesel exhaust exposure induced CYP1A1 expression and AHR activation without a coordinated antioxidant response 221. There is a stronger relationship between NRF2 expressions and its related antioxidant response with osteoclasts than osteoblasts. The inhibition or activation of NRF2 signaling by ML385 (an NRF2 inhibitor) or curcumin (an NRF2 activator), respectively, modulated ROS levels, which affected the function of osteoblasts and osteoclasts. The inhibition of NRF2 enhanced osteoclast genesis, whereas its activation suppressed it. By contrast, osteogenesis decreased irrespective of whether NRF2 was inhibited or activated. These findings highlight the distinct ways in which the NRF2-mediated antioxidant response regulated osteoclast and osteoblast differentiation 222. Additional studies are required to determine the molecular link between bone genesis and the NRF2-ROS axis.

*Jdp2* KO mice exhibited osteopetrosis resulting from impaired osteoclast genesis, and their neutrophils were morphologically normal, but impaired surface expression of Ly6G, bactericidal function, and apoptosis. *Jdp2* KO mice were highly susceptible to *S. aureus* and *Candida albican*s infection. Thus, JDP2 plays an important role in bone homeostasis and host defense by regulating osteoclast and neutrophil differentiation 148,223.

Chromatin remodeling and epigenetic regulation were evident of AHR-NRF2-JDP2 complex. The BRG1/BRM-associated factor complex was identified as another complex that interacted with AHR or JDP2 or NRF2 (data not shown) 224,225. AHR directly interacted with BRG1 224 but did not associate with the enhancer elements in ARNT-deficient cells; thus, the AHR-ARNT complex was critical for forming a complex with BRG1 226. Moreover, IL-6 expression was dependent on AHR and BRG1 activity 227. The upregulation of lymphoid-specific helicase/SMARCA6 activated AHR signaling during lung cancer progression 228. BaP increased the expression of lymphoid-specific helicase/SMARCA6, which had lymphoid-specific helicase activity and played a crucial role in epigenetic regulation by modulating DNA methylation and chromosomal remodeling.

Possibly, the AHR-ARNT dimer also activated the transcription of target genes by recruiting various transcription cofactors, including CBP/p300 229, steroid receptor coactivator 1 (SRC1)/NCOA1, SRC2/p160/bHLH-PAS, NCOA2/glutamate receptor-interacting protein 1/transcriptional intermediate factor 2, and SRC3/CBP/p300/cointegrator-associated protein 1 (p/CIP)/AIB/ACTR/RAC/GTRAM-1 230,231; coactivator-associated arginine methyltransferase 1 and protein arginine methyltransferase 1; and ATP-dependent chromatin remodeling components including BRG1 232. After chromatin opening, the cell cycle initiation factor genes were initiated into transcription by the recruitment of the Pol II transcriptional initiation complex. In the nucleus, AHR synergized with RB to repress early region 2 binding factor (E2F)-dependent transcription and induced cell cycle arrest 233. Moreover, activated AHR formed AHR-E2F1 protein complexes to block E2F1-dependent gene expression and apoptosis 234.

In addition to the chromatin modifier function of JDP2, HDAC inhibitors, such as butyrate or SCFAs, promoted the recruitment of AHR to the *CYP1A1* promoter in human Caco-2 cells and HepG2 cells 235. JDP2 was a Myc-interacting and TP53-suppressing gene and was activated by the induction of HDAC1/2, which was required for the survival of JDP2-overexpressing lymphoma 236. The JDP2-ATF3 heterodimer reportedly interacted with a series of HDAC members, including HDACs 1-6 and 10. The association of HDAC3 and HDAC6 with JDP2 and ATF3 occurred via direct protein-protein interactions. Only part of the N-terminal bZIP motif of JDP2 and ATF3 basic domain was necessary and sufficient for the interaction with HDACs in a manner that was independent of coiled-coil dimerization 66,114,115,121. JDP2 was also associated with other proteins involved in chromatin regulation, such as Jumonji C histone demethylase 1B, mitogen-activated protein kinase kinase 6, Glis family zinc finger 1, NANOG, estrogen-related receptor beta, and Spalt-like transcription factor 4, that reprogram MEFs to iPSCs 145,236,237.

In recent decades, AHR-NRF2 has been recognized as a critical modulator of disease because of the role of the AHR-NRF2 pathway in the regulation of the redox system and inflammatory responses for homeostasis 144,192. Recent studies have clarified how the AHR-NRF2 axis coordinates with chromatin regulators such as the histone chaperone JDP2. Studies on the pathophysiology of the AHR-NRF2-JDP2 axis will provide key insights into the modulation of the phase I and II enzyme systems to maintain ROS homeostasis for cellular protection. Here, we propose that JDP2, which is a histone chaperone, acts as a bridge between chromatin modulators and both open and closed chromatin, guiding the RNA polymerase complex to the AHR-NRF2 gene battery. An animal-free *in vitro* model, such as organoid-on-a-chip and organ-on-a-chip, should be generated to replace organoid models for screening therapeutics and preclinical studies 238,239.

## Conclusions

In the present review, the newly described AHR-NRF2-JDP2 gene battery provides evidence that JDP2 contributes to the association of the AHR-NRF2 battery with *AHR* promoter activation and ROS homeostasis. The AHR-NRF2-JDP2 gene battery is extremely sensitive and can be activated by phase I enzyme ligands, such as TCDD and BaP or tryptophan derivatives. Oxidative stress is greater in the steady state in JDP2-deficient MEFs than in wild-type MEFs. Phase I enzyme ligands induce activation of the *AHR* promoter and play roles in the phase II enzyme-encoded promoter through the phase II transcription factor NRF2 and the chromatin modifier JDP2. The AHR-JDP2 and NRF2-JDP2 complexes are recruited to the DRE region first and then to the ARE region of the *AHR* promoter to activate gene expression. Thus, these proteins are critical for modulating the ROS balance, and JDP2 modulates the balance between detoxification and antioxidation responses. The activation of phase I enzymes by binding of the AHR-NRF2-JDP2 complex to the DRE results in a significant increase in ROS. After ROS accumulates to a threshold level, it induces the AHR-NRF2-JDP2 complex on the DRE cis-element, and then the complex binds to the ARE to regulate the maintenance of homeostasis against oxidative stress. The newly described AHR-NRF2-JDP2 gene battery links the AHR-JDP2 and NRF2-JDP2 axes. Therapeutics are being developed to target this new cascade AHR-NRF2-JDP2. As of 2024, 115 interventional human PSC (hPSC) trials with regulatory approval have been performed and 83 hPSC products have been developed. Most of these trials focused on the eye, central nervous system, and cancer treatments. To date, more than 1,200 patients have been treated using hPSC products, accounting for more than 1,011 clinical administrative cells 240.

## Figures and Tables

**Table 1:** Summary of chromatin regulation of AHR and AHR target gene products

Modification	Details	Reference
[DNA methylation and demethylation]		
(AHR promoter)		
DNA methylation in CpG island	Low levels of DNMT1, 3a, 3b & MBP2 and SP1 recruitment in AHR promoter in ALL enhances AHR promoter activation.	20
	DNMT inhibitor (Zebularine) induces DNMT1, 3a, 3b reduction and AHR upregulation (in ALL cells; ReH cells; Jurkat cells).	46
	AHR promoter -- SP1 in CpG islands are active in MCF7. DNA adduct (2-amino-1-methyl-6-phenylimidazo[4,5-b] pyridine) mediated H3K27me3 reduction in CpG of AHR promoter is critical in long term estrogen exposed (LTEE) MCF7 breast cancer cells.	47
DNA methylation	Rheumatoid arthritis associated hypermethylation of Ahr promoter in mice -- DNMT inhibitor (5-AzaC) induced AICDa reduction - thus, it reduces class-switch recombination. This process leads to diminished IgG1 production and amelioration of autoimmune arthritis.	55
(BRCA promoter) DNA methylation	In TNBC, overexpressed AHR induces epigenetic silencing of *BRCA1 promoter by* transcriptional activation of estrogen receptor (ER)α. GEN (Genistein) suppressed AHR dependent *BRCA1 promoter* hypermethylation (CpG islands), and the restoration of ERα-mediated response in HCC38 cells (TNBC with hypermethylated BRCA1 locus). ERα in HCC38 cells or MCF7 cells -- BRCA activation is induced by decreased CpG methylation and then AHR recruitment to BRCA locus.	48
DNA methylation	Resveratrol committed the reversed epigenetics changes and AHR binding to BRCA promoter in breast cancer cells.	101
DNA methylation	TCDD induced BRCA promoter-hypermethylation/silencing by methyl marks included MDB2, H3K9me3, DNMT1, 3a and 3b.	49
(FOXP3 promoter) (IL-17 promoter) DNA methylation	CpG islands -- decrease FOXP3→ Treg function is decreased to CD4+CD25^-^ -- increased Th2 phenotype in mice. Activation of T cells from AhR^(+/+)^ but not AhR^ (-/-)^ mice, in the presence of TCDD, promots increased differentiation of Treg while inhibiting Th17 cells. Analysis of MLN or LP T cells during colitis revealed increased methylation of CpG islands of Foxp3 promoter and demethylation of IL-17 promoter, which was reversed following TCDD treatment.	50,51
(CYP1A1 promoter) DNA methylation	Dioxin-AHR dependent DNA demethylation in CYP1A1 promoter via Tdg (Thymine DNA glycosylase) in mouse liver. AHR, Tdg, Tet2, Tet3 are required for TCDD induced DNA demethylation.	54
[Acetylation and deacetylation]		63,86
ARNT moiety ARNT - CBP/p300	p300/CBP induced acetylation of ARNT in mouse 293T cells and Yeast system.	61,62
(AHR promoter)		
HDAC1 inhibitors (TSA, n-Butyrate)	HDAC1 inhibitor (TSA, n-Butyrate) activate AHR/ARNT transcription in mouse osteoclasts RAW264 cells, or rat bone marrow cells.	241
HDAC1 inhibition & RhoA activation (3-Methylcholanthrene, Simvastatin)	3-Methylcholanthrene (3-MC) induced AHR activation in human renal cell carcinoma -- EMT activation-tumor marker expression in human renal epithelial cells (hREC and RCC) cells. Simvastatin inhibits 3MC induce tumor induction by reducing HDAC1 and RhoA upregulation in RCC cells	80
HDAC8 inhibitor	HDAC8 inhibitor (PC1-34051) in allergic asthma model, mouse lung cancer cells Raw 214. 7 cells. Amelioration of AHR expression and airway inflammation and macrophage M2 polarization	242
HDAC inhibitors (n-Butyrate)	Butyrate acts as iHDAC leading to an increase in recruitment of AHR to the target gene promoter in the presence of tryptophan-derived AHR agonists. The data contribute to a novel understanding of the complex regulation of AHR activation by gut microbiota-Tryptophan derived metabolites in mice	77
HDAC I/IIb inhibitor (Purinostat Mesylate)	Human Ph+ leukemia cells and CD34+ leukemia from CML patients (leukemia stem cells; LSC) repress c-Myc, β-catenin, E2F, EZH2, Alox5, mTOR injectable formulation of PM (PMF)- increased glutamate metabolism in LSCs by increasing glutaminolysis inhibition. Combination of PMF and glutamate inhibitor (BPTES) synergistically eradicate LSCs by altering multiple key proteins and signal pathways of LSC survival and self-renewal. A new strategy for eliminating LSCs (by targeting HDAC I/IIb and glutaminolysis) -- potentially provide guidance for PMF clinical trials for TKI resistance CML patients	85
HDAC inhibitor (SB939; pracinostat) plus AHR agonists	Arresting of mouse experimental autoimmune encephalomyelitis (FAE) through STAT3 acetylation by IL6 in the stable transcriptional activation of indoleamine 2,3-dioxygenase 1 (IDO1) gene. The therapeutic effect of SB939 also requires the AHR, which is expressed mainly in CD4^+^ T cells and macrophages in CNS disease lesions.	86
Polycyclic Aromatic Hydrocarbons (PAHs) AHR agonists	PAHs treatment in mice; *Lactbacillus murinus* alleviates lung inflammation (SCFA) induced by PAHs in mice - Gut, Lung tissues; IgE, IL-4 and IL-17A in bronchoalveolar lavage fluid (BACF) fluids. AHR, Cyp1A1, Foxp3, HDAC activity are increased; AHR increasing causes Th17/Treg imbalance--- IA/IA2a in serum	87
HDAC inhibitors (Na-butyrate and curcumin)	Na-butyrate and curcumin result in reduction of asthma severity via HDAC1 inhibition in mice. HDAC1, HIF-1a, VEGF, p-AKP, p-PI3K are reduced by treatment with curcumin and Na-butyrate. p-p38, IL5, GATA3 are also reduced. p-AKT/p-PI3K/HIF-1a/VEGF axis is critical for air inflammation in mice.	89
AHR agonist (Indoxyl sulfate =IS)	IS induces AHR synthesis and oxidate DNA damage by reduction of AHRR, Cyp1a, SIRT3, SIRT7-- affects bone mineral production in rat.	90
AHR agonist (Cinnabarinic acid = CA)	CA results the stanniocalcin 2 (STC2) upregulation as AHR target gene-- cytoprotection--- ER, ROS stress induces apoptosis in mice. CA but not TCDD induce STC2 induced MTA2 (metastasis-associated protein 2) = CA dependent MTA2 to STC2 promoter to induce H4K acetylation (H4Kac) and cytoprotection	243
(Cyp1a1 promoter)	Bap induction-Cyp1a1 promoter bound HDAC1 is released in mouse Hepa1 cells-Cyp1a1 activation-H3K4me over H3K27me, H3S10 phosphorylation - Cyp1a1 transcription activation. *Cyp1a1* induction by the AHR/ARNT is associated with modification of specific chromatin marks, hyperacetylation of H3K14ac and H4K16ac, H3K4me3, and H3S10 phosphorylation. HDAC1 and DNMT1 form complexes on the *Cyp1a1* promoter of uninduced cells but HDAC1 inhibition alone is not sufficient to induce *Cyp1a1* expression, although it allows for the hyperacetylation of H3K14ac and H4K16ac to levels similar to those found in BaP-induced cells.	244
Phase I enzyme ligands in Cyp1a1 promoter	AHR-NFκB p65 interaction induce pCY1A1 histone epigenetic changes in mouse hepa1c1c7 cells, African green monkey kidney fibroblast-like Cos 7 cells. H4K5ac and demethylation of H4K3 marks.	245,246
(LTBP-1 gene promoter) HDAC2 and pCREB (S133-P)	Latent TGFβ-binding protein-1 (LTBP-1) as the TGFβ target is critical for the activation in the extracellular matrix of mice--AHR regulates Ltbp-1 transcription by a mechanism involving recruitment of co-activators such as CREB1 and co-repressors such as HDAC2 to the Ltbp-1 promoter. AHR expression is repressed Ltbp-1 promoter activation by HDAC2 binding in WT MEFs but in AHR^-/-^ MEF HDAC2 and pCREB (Ser 133-P) are decreased and Ltbp-1 transcription is reduced.	88
[Chromatin Modifiers]		
Med220-Cyp1a1 promoter TRAP/DRIP/ARC/Mediator complex	TCDD induces Cyp1a1 gene activation by Med220. CDK8 and TRAP/DRIP/ARC/Mediator, P300 and p/CIP are required in Hepa1 cells	110
Med1, CTCF and AHR	Liver biopsy specimens of patients with acute liver failure (ALF). Liver specific miR-122 knockout (LKO) mice in acetoaminophen induced Cyp2e1 and Cyp1a2 genes; acetoaminophen or N-acetyl-p-benzoquinone in mice. In miR-122 knockout LKO mice, Cyp1a2 gene is upregulated-- AHR and CTCF, and Med 1 are upregulated Human Hepa RG cells--miR122 depletion induces differentiation. miR-122 plays a role for acetoaminophen induced detoxification	111
BRG1-AHR/ARNT promoter	BRG-AHR-ARNT promoter to increase Cyp1A1 gene activation TCDD induces AHR-ARNT activation to CYp1a1 gene activation-- BRG1 potentiates AHR/ARNT reporter genes in TCDD induced Hepa1c1c7 cell. BRG1 induces AHR/ARNT reporter genes upregulation in SW13 and C33A cells. Glutamine rich domain of AHR interacts with BRG1 mediator molecule.	224
BRG1-AHR-Cyp1a1 promoter	BRG1 is an AHR coactivator to recruit to CYP1A1 promoter in mouse hepatocytes and human retinal pigment epithelial cells (ARPE-19 cells) --CYP1A1 gene promoter -12 kb upstream enhancer is the target of BRG1-AHR complex recruitment.	225
BRG1-AHR-IL6 promoter	Head & neck squamous cell carcinoma (HNSCC) lines -- cytokine producing tumor with IL6, constitutively bound AHR at IL6 promoter, allowing for higher inducible *IL6* transcription. AHR antagonist led to dismissal of the AHR from the *IL6* promoter and recruitment of corepressor complexes, thus diminishing cytokine expression. siBRG1 shows the similar activities.	227
SMARCA6/HELL-AHR promoter	BaP exposure induces SMARCA6 (SWI/SNF2-Related, Matrix-Associated, Actin-Dependent Regulator of Chromatin, Subfamily A, Member 6) expression in NSCLC (Non-small-cell lung carcinoma) to activate AHR signaling and DNA methylation and chromosomal remodeling.	227
(TCDD- SRC/NCoA-2, p/CIP -interacted with AHR- CYP1a1 enhancer)	TCDD activates AHR-ARNT luciferase by coupling the cofactor SRC-1/NCoA-1, NCoA-2/GRIP-1/TIF-2, and p/CIP/AIB/ACTR which is interacted with AHR to enhance the CYP1a1 enhancer in mouse Hepa1c1c7 cells. SRC-1 and NCoA-2 but not p/CIP are capable of interacting with ARNT in vivo after transient transfection into mammalian cells, while AHR is capable of interacting with all three coactivators SRC-1, NCoA-2, p/CIP. Interactions of ARNT and AHR with SRC-1 with immunocytochemical techniques. Furthermore, SRC-1, NCoA-2, and p/CIP all associate with the CYP1A1 enhancer region in a TCDD-dependent fashion, as demonstrated by chromatin immunoprecipitation assays.	229
(SRC1-AHR or PIP140 with AHR in response to TCDD)	SRC1 in mouse Hep1c1c7 cells (hepa-1 cells) proximal of p300/CBP interaction dimer -- SRC1-p300/CBP interaction. SRC-1 Q rich domain interacts with AHR (TA domain), but not ARNT AhR transactivation domain is sufficient for enhanced coactivation mediated by SRC-1 in the presence of a transactivation domain deleted ARNT protein.	230
TCDD-AHR-CPS1 to H1 citrullination	TCDD-AHR recruited CPS1 to NC-XRE of PAI-1 promoter to generate HIK34hcit. H1.4K34 acetylation by GCN5 in spermatogenesis is critical.	102
(NRF2 acetylation) NRF2-CBP/p300	CBP (C/H3 domain) interacts with NRF2 Neh4 and Neh5 domain and acetylates NRF2, NRF2 18K site might be crucial for p300 acetylation mainly. Clinical-grade CBP/p300 inhibitor CCS1477 represses the global NRF2-dependent cytoprotective transcription program.	67-76, 225
NRF2-Med16	NRF2-Med16 complex is detected.	112
(JDP2- HAT/HDAC)	JDP2 is INHAT of p300/ CBP coactivator	36
	JDP2 recruits HDAC3, and HDAC1, 2, 4-6,10	114,121,122
JDP2-PRMT5	JDP2-PRMT5 elicit H3R2me1/H3R2me2 induced transactivation via TCF independent pathway by recruitment of WD repeat domain 5 (WDR5)/myeloid/lymphoid or mixed-lineage leukemia protein (MLL) methyltransferase complexes.	247
JDP2-Sall4-NuRD	Sall4, Jdp2, Glis1 and Esrrb (JGES) can reprogram MEFs to iPSCs efficiently, but only Sall4 is indispensable capable of recruiting endogenous components of NuRD. Sall4 recruits NuRD complex to open chromatin in MEFs to ensure the closure of somatic loci. This recruitment is dependent on the N-terminal motif of Sall4 and can be transferred to an unrelated factor such as Jdp2.	145,236,237
TIP60-UHRF 4K acetylation- JDP2	Acetylation of UHRF1 4K residues by TIP60 is important for colon cancer cell growth. Furthermore, upregulated JDP2 expression by acetylation-deficient mutant of UHRF1 might be an important epigenetic target for colon cancer cell proliferation.	108
SUMOylation-JDP2	JDP2 is a candidate for SUMOylation and SUMOylation affects JDP2-mediated Mc2r transcriptional activity in mice.	248

## Abbreviations

AD: Atopic dermatitis
AHR: aryl hydrocarbon receptor
AP1: activation protein 1
ARE: antioxidation response element
ARNT: aryl hydrocarbon receptor
ATF: activation transcription factor
BAF: BRM-associated factor
Bap: benzo[a]pyrene: BRCA
breast cancer: BRG1: brahma-related gene 1
cAMP: cyclin adenosine monophosphate
CBP: cAMP response element binding protein-binding protein
CKD: chronic kidney disease
CoQ19: Coenzyme Q10
CYP1A1: cytochrome P450 family 1 subfamily A member 1
CYP1B1: cytochrome P450 family 1 subfamily B member 1
DIC: doxorubicin-induced cardiotoxicity
DIM: 3,3′-diindolylmethane
DKO: double knockout
DNMT: DNA methyltransferase
DRE: dioxin-response element
E2F: early region 2 binding factor: Essrb: estrogen related receptor, beta
FICZ: 6-Formylindolo [3,2-*b*] carbazole
FLG: filaggrin
FOXP3: forkhead box P3
FSP1: ferroptosis suppressor protein 1
Glis1: Glis family zinc finger 1
GPX4: glutathione peroxidase 4
HAT: histone acetyltransferase
HDAC: histone deacetylase
HMOX1: heme oxygenase 1
IAA: Indole 3-acetic acid
I3C: Indole-3-carbinol
Id1: inhibitor of DNA binding 1
IDO: indoleamine 2,3-dioxygenase
IECs: intestinal epithelial cells
ILA: indole-2-lactic acid
iPSC: induced pluripotent stem cells
IS: indoxyl sulphate
IVL: Involucrin
JDP2: Jun dimerization protein 2
Jhdm1b: Jumonji C histone demethylase 1B
KDM2B: lysine demethylase 2B
KLF6: Krüpple -like factor 6
Kyn: Kynurenine
LDNP: *Lactobacillus rhamnosus* GG (LGG)-derived exosomes like nanoparticles
LOR: loricrin
LTBP-1: late transforming growth factor-β-binding protein 1
LPS: lipopolysaccharide
Lrh1: liver receptor homolog 1
MALSD: metabolic dysfunction-associated steatosis liver disease
3-MC: 3-methylcholanthrne
MEF: mouse embryonic fibroblast
MIP2α: macrophage inflammatory protein 2α
MKK6: mitogen-activated protein kinase kinase 6
MTA2: metastasis tumor-associated protein 2
NAD(P)H: nicotinamide (phosphate) dehydrogenase
NCOA1: nuclear receptor coactivator 1
NET: neutrophil extracellular trap
NFkB: nuclear factor-kappa B
NLRP3: NLR family pyrin domain containing 3
NR5A2: nuclear receptor subfamily 5, group A, member 2
NRD: nucleosome remodeling and deacetylase
NRF2: nuclear factor-erythroid 2-related factor 2
NSCLC: non-small cell lung cancer
4-OH-2: 4-Hydroxy-2-nonenal
PAHS: Polycyclic aromatic hydrocarbons
PAS: Per-Arnt-Sim domain
p/CIP: cointegrator-associated protein
PI3K: phosphoinositide 3-kinase
Pkm2: pyruvate kinase muscle in form 2
ROS: reactive oxygen species
POL II: RNA polymerase II
RHOA: RAS homolog family member A
SALL4: Spalt-like (SALL) gene family 4
SCFA: short chain fatty acids
SFN: sulforaphane
sMAF: small musculoaponeurotic fibrosarcoma
SMARCA4: SWI/SNF2-related, matrix-associated, actin-dependent regulator of chromatin, subfamily A, member 4
SRC1: steroid receptor coactivator 1
SWI/SNF: switch/sucrose non-fermentable
TECs: thymic epithelial cells
TET: ten-eleven translocation
TKO: triple knockout
Trp53: tumor suppressor protein 53
IECs: intestinal epithelial cells
UHRF1: ubiquitin-like with prolyl hydroxylase domain and RING finger domain 1
XRE: xenobiotic response element
T-ALL: T cell acute lymphoblastic leukemia
TCDD: 2,7,8-tetrachlorodibenzo-p-dioxin
TRAMP: transgenic adenocarcinoma of the mouse prostate
